# A Systematic Literature Review on Integrated Deep Learning and Multiagent Vision-Language Frameworks for Pathology Image Analysis and Report Generation

**DOI:** 10.34133/csbj.0023

**Published:** 2026-04-13

**Authors:** Usama Ali, Imran Shafi, Jamil Ahmad, Arlette Zarate Caceres, Thania Candelaria Chio Montero, Hafiz Muhammad Raza ur Rehman, Imran Ashraf

**Affiliations:** ^1^ College of Electrical and Mechanical Engineering, National University of Sciences and Technology (NUST), Islamabad 44000, Pakistan.; ^2^ Department of Computing, Abasyn University-Islamabad Campus, Islamabad, Pakistan.; ^3^ Abdul Wali Khan University, Mardan, Pakistan.; ^4^ Universidad Internacional Iberoamericana, Campeche 24560, Mexico.; ^5^ Universidade Internacional do Cuanza, Cuito, Bie, Angola.; ^6^ Universidad de La Romana, La Romana, Dominican Republic.; ^7^ Universidad Europea del Atlantico, Santander 39011, Spain.; ^8^ Universidad Internacional Iberoamericana, Arecibo, Puerto Rico 00613, USA.; ^9^ Department of Information and Communication Engineering, Yeungnam University, Gyeongsan-si 38541, Republic of Korea.

## Abstract

This systematic literature review investigates the integration of deep learning (DL), vision-language models (VLMs), and multiagent systems in the analysis of pathology images and automated report generation. The rapid advancement of whole-slide imaging (WSI) technologies has posed new challenges in pathology, especially due to the scale and complexity of the data. DL techniques in general and convolutional neural networks and transformers in particular have substantially enhanced image analysis tasks including segmentation, classification, and detection. However, these models often lack generalizability to generate coherent, clinically relevant text, thus necessitating the integration of VLMs and large language models (LLMs). This review examines the effectiveness of VLMs and LLMs in bridging the gap between visual data and clinical text, focusing on their potential for automating the generation of pathology reports. Additionally, multiagent systems, which leverage specialized artificial intelligence (AI) agents to collaboratively perform diagnostic tasks, are explored for their contributions to improving diagnostic accuracy and scalability. Through a synthesis of recent studies, this review highlights the successes, challenges, and future directions of these AI technologies in pathology diagnostics, offering a comprehensive foundation for the development of integrated, AI-driven diagnostic workflows.

## Graphical Abstract

**Figure:**
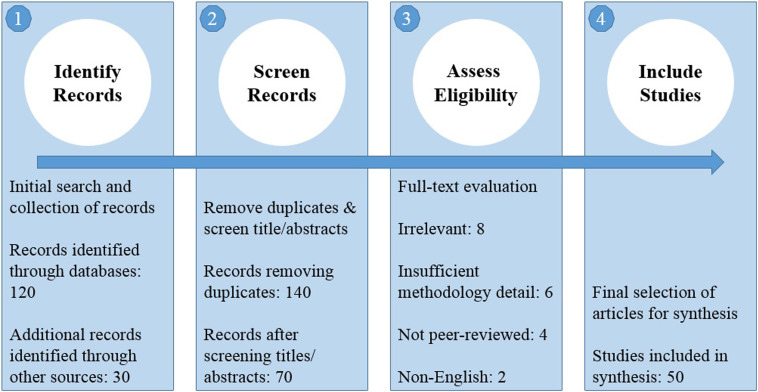

## Introduction

Pathology is a cornerstone of medical diagnostics, offering microscopic insight into tissue samples that reveal crucial information about disease presence, progression, and prognosis [1]. Traditionally manual inspection of glass slides under a microscope represents an expensive, cumbersome, and unreliable diagnostic technique that is both time-consuming and subjective because of interobserver variability. These techniques represent a step change toward whole-slide imaging (WSI) technology that enabled entire tissue slides to be digitized at high resolution. Digital slides allow ready sharing of data across institutions and enable telepathology and collaborative diagnostics based on greater flexibility of viewing tissue morphology [2].

The scale of pathology image data has exploded with the adoption of WSI in clinical and research environments, generating millions of gigapixel images annually. As a result of this unbridled growth, however, it has also brought about some critical challenges, namely, the volume of images that require interpretation along with the complexity of visual information contained within [3]. Automated and scalable image analysis methods are in high demand to reduce pathologist workload, minimize human error, and reduce diagnostic delays critical in diseases such as cancer where early intervention is crucial [4].

### Role of deep learning in medical imaging

Deep learning (DL), especially convolutional neural networks (CNNs), has revolutionized the field of medical image analysis. DL enables automatic extraction of hierarchical features directly from image data [5]. CNNs have been shown great success in cell detection, tumor classification, and tissue segmentation problems in pathology, sometimes achieving accuracy on par with or surpassing human experts. These models are very good at recognizing subtle patterns in the localized sense but have traditionally struggled with understanding the full tissue context, which is vital to adopting a holistic diagnostic methodology [6].

Recent advances have introduced transformer-based architectures, which offer superior capabilities to model long-range dependencies and global context, addressing some limitations of CNNs. New hybrid models improving the performance of CNNs and transformers try to benefit from the best of both worlds, acquiring fine-scale cellular details while welding large-scale tissue structures [7]. Although these innovations address some of these issues, the whole slide image size remains enormous, staining variation is still a problem between laboratories, and robustness to diverse datasets is critical for moving to real-world clinical deployment [8].

### Emergence of vision-language models and large language models in medical report generation

While DL models have greatly enhanced the visual analysis of pathology images, translating these complex visual patterns into meaningful, clinically coherent text reports poses a distinct challenge. Pathology reports need not only to describe findings precisely but also to put them in context, using clinical guidelines and terminologies, for clinicians to make informed decisions.

The rise of large language models (LLMs) provides a promising solution to automate this translation process. Similarly, vision-language models (VLMs) fuse image understanding and natural language processing (NLP), resulting in descriptive captions, diagnostic summaries, and complete reports consistent with clinical standards [9]. In this area, there has been some significant progress in radiology, with models producing initial diagnostic reports based on x-rays and computed tomography (CT) scans. However, WSIs are special in that pathology exhibits unique complexities of higher resolution and higher intricacy and that WSIs must span both fine and coarse image features [10]. These complexities are only just beginning to be taken up in current research, but are not answered by unified systems that effectively and reliably couple image analysis and text generation for pathology.

### Need for integrated multiagent systems

Conventional artificial intelligence (AI) systems are commonly applied in single tasks within medical imaging like classification or generation of reports. Nevertheless, they are unable to produce whole-potential diagnostic processes essential to clinical decision-making process because of the operations like normalization and thresholding that occur in the siloed approach [11]. An interesting solution is the multiagent method that involves the use of several domain-specific AI agents to work together to perform multifaceted correlated tasks.

Multiagent systems (MAS) allow agents of specific expertise to be specified, one that produces detailed image features, one that produces preliminary textual descriptions, and one that checks and corrects outputs according to medical ontologies and clinical guidelines. Part of this team work is iteration: the agents communicate to increase the caliber and clinical validity of the end-result pathology report. The distributed character of MAS make them prone to scalability and flexibility, which allows the addition of new agents to address new emerging challenges or new specific clinical requirements. This kind of integration can revolutionize the pathology operations by automating complete end-to-end workflows, removing human errors, and generating annotated datasets that can be used to accomplish research studies and development applications [12].

### Objectives and scope of the systematic literature review

The purpose of this review is to critically examine the convergence between DL and MAS and LLMs on pathology image analysis and report generation. The architectures of the models, datasets, and training processes associated with the automated interpretation and reporting of pathology images will be reviewed, and clinical uses will be discussed in the wider scope of literature. The special attention will be drawn to the combined MAS that are based on the vision language models; they are the state of the art in the field.

Through a systematic evaluation of the existing literature, this review aims to determine effective methodologies, evident gaps, and crucial gaps in research. It will also look into the ways in which these technologies are being translated to clinical practice regarding scalability, interoperability, and ethics issues. An aggregate outcome of the review will be taken as a holistic foundation to the presented hybrid multiagent design to continue developing it as a useful practical application in pathology diagnosis.

It is critical to explicitly define the scope of this review, which centers on Pathology WSI. Pathology WSI tasks involve the analysis of gigapixel, microscopic cellular images that require multiscale zooming and navigate extreme morphological complexity. This is different from radiology work (e.g., CT, MRI, or chest x-rays), which typically concerns macroscopic, anatomic structures at far lower resolutions. In general, though, this review contains a selective collection of more recent radiology based studies, especially those on VLMs and natural language generation (NLG). These nonpathology structures are essential architecture blueprints since radiology AI arose earlier in the field of automated report construction and multiagent coordination. To achieve the aims of this review, there is a need to analyze the pioneer systems of radiology because their approach to cross-modal alignment and agentic orchestration currently is being translated to address the unique, high-resolution problems of digital pathology.

### Research questions

The systematic literature review (SLR) will be guided by the following key research questions:

• What are the current state-of-the-art DL approaches used in pathology image analysis, and how do they address challenges such as high-resolution data and dataset variability?
• How have VLMs and LLMs been employed to generate clinically relevant text from medical images, particularly in pathology?
• What roles do MAS play in integrating image analysis with text generation in medical diagnostics, and what are the architectures and strategies that facilitate effective interagent collaboration?

## Related Work

The integration of AI in healthcare, particularly in medical imaging, has become one of the most transformative technological advances in modern medicine [13]. A potential application of AI systems, particularly DL algorithm-based, reveals an ability to greatly improve diagnostic accuracy, automate workflows, and improve clinical decision-making [14]. Their applications in medical imaging have produced some very encouraging results in improving the early detection of disease and streamlining treatment planning across a range of imaging modalities including those in radiology, pathology, and dermatology [15–17]. This integration is, however, a significant challenge, as it is inherently a multifaceted task involving multiple types of data in the healthcare space, variability of imaging data, and population diversity [9].

Medical imaging or the interpretation of images of human body is a very significant aspect of diagnostic medicine. Images provided by assorted medical resources, including endoscopy, tissue pathology sections, and scans, including CT, x-rays, positron emission tomography, and MRI, can be analyzed and used to identify many diseases [18]. Last but not least, DL models, particularly CNNs and vision transformers, have formed the foundation of such change that helps the segmentation, classification, and detection of abnormalities on a more precise level become possible. Nonetheless, even though they are now skilled pictorial analysts, more needs to be done in linking these DL models with additional ancillary datasets accessible in the field (electronic health records [EHR]) of clinical information [19].

Regardless of the improvements in AI, the field is still characterized by a number of weaknesses, such as generalization problems, model explainability, and interpretation of findings in a clinical setup [20]. Nonetheless, the constraints are enhanced when it comes to multicenter, multidevice use of AI models, where imaging devices, data collection mechanisms, and patient demographics may influence model performance as well all at once [21].

### DL and its applications in medical imaging

DL has quickly become a key tool in medical imaging, especially for tasks like diagnosing diseases, segmenting organs, and detecting abnormalities [21]. One of the most notable benefits of the DL models, including CNNs, is the automatic phrasing of hierarchical features of medical images, which is historically a highly tedious and manual procedure performed by operational professionals in the field [14]. This capability has led to dramatic advances in the accuracy of diagnostics, even surpassing the readout capability of human radiologists in some directions, sometimes, as noted, by a wide margin in some areas [19]. Nonetheless, with CNN-based models, they have been established to attain a high diagnostic accuracy of diabetic retinopathy (area under the curve [AUC] = 0.933 to 1), lung cancer (AUC = 0.864 to 0.937), and breast cancer (AUC = 0.868 to 0.909) [14]. To accomplish this, they are trained on massive large quantities of data and taught to identify these very low-level patterns in medical images, which human experts could not do with repeated trials. Although the achievements here are great, the DL in medical imaging with the aim of improving the care received by the patients does have its challenges. Nevertheless, the fact that these models are affected by changes in the dataset and fluctuate when there is a change in imaging protocol, type of the scanner, and patient demographics is one of the biggest limitations of these models [18]. Such inconsistency among clinical settings causes the deployment of such models to reduce model performance. An example illustrating this can include differences between the approaches that the hospitals employ in amassing data, which can restrict the use of the model to fresh settings [19]. Data augmentation, synthetic data generation, and domain adaptation have been considered to address these issues through enhancing the strength and applicability of the DL models in medical imaging practice [22].

Another important aspect of DL in medical imaging is the use of data augmentation techniques. Data augmentation is the process of making additional training data by transforming the original dataset with applications such as rotations, scaling, and flipping [22]. In medical imaging, as is often the case with annotation, we lack or have limited annotated data that are costly to obtain, which makes this technique valuable [22]. Data augmentation artificially enlarges the dataset to help improve the model’s performance in a scenario where the number of labels is limited. In addition, medical imaging applications have attracted interest in the subfield of machine learning known as few-shot learning, in which models are trained with very little labeled data as training data [23]. To tackle an insufficient amount of annotated medical images, we introduce a few-shot learning methods that enable models to generalize from a few examples. Studies have demonstrated that these techniques can improve the performance of DL models on different medical imaging tasks, e.g., tumor detection and organ segmentation [21]. Although they may be promising, applying data augmentation and few-shot learning remains challenging in clinical settings. Therefore, to avoid poor accuracy caused by noisy augmented images, the quality of augmented data is also important. Second, given that few-shot learning presents a promising approach, it in general requires specialized algorithms and rigorous validation to be reliable in practical medical applications [23].

In multicenter or multidevice studies, image harmonization is critical to ensure that models trained on data from different sources can generalize effectively across varied clinical environments [23]. Inconsistencies in such model performance can arise due to variations in imaging equipment, patient demographics, and acquisition protocols [21]. To standardize medical images, often image harmonization techniques (such as grayscale normalization and image resampling) are employed to make the images more consistent and comparable within and across a variety of image centers and devices [19]. Image harmonization has been shown recently to be highly effective in increasing the diagnostic performance of DL models. In one example, in multicenter breast cancer studies of breast cancer detection, image harmonization improves the classification accuracy by up to 24.42% [21]. Furthermore, authors demonstrate that color normalization techniques can slightly improve AUC scores on external test sets and may serve as a way to improve robustness to multicenter data used in training [14].

### VLMs in medical imaging

VLMs have emerged as a powerful tool in medical image analysis by integrating both visual and textual information. Bringing the strength of CV and the strength of NLP, VLMs allow AI systems to process medical images with accompanying textual information [24]. Medical report generation is one of the main usage areas of VLMs in healthcare, as the model generates textual descriptions of ratings after analyzing medical images. The value of this task is especially high when applied to radiology, which suffers from an extremely large amount of imaging data that creates a barrier to manual detailed report writing for every case. VLMs have recently been shown to generate text that is coherent and relevant to the medical domain when used for automating medical report generation [25]. For example, VLM has been used to automatically generate reports from chest x-rays and CT scans, where the reports are given to radiologists to summarize findings in aiding the diagnosis and treatment planning. They have been shown to increase efficiency and help alleviate the work of healthcare professionals. Furthermore, VLMs have been deployed as solutions to visual question answering (VQA) problems, explicitly, answering clinical questions given medical images, thereby improving clinical decision-making [9].

While VLMs excel at processing visual data, integrating medical imaging with other forms of clinical data, such as EHRs, is essential for improving diagnostic accuracy and treatment planning [19]. Patient information that is vital to the compositional context for the interpretation of medical images, including medical history, lab results, and medication details, are all contained in EHRs. By integrating these data with image analysis models, newly generated AI models can make diagnoses and treatment recommendations that equally consider imaging data as well as the patient’s clinical background [26]. New advancements in DL-based data fusion techniques have also been growing to integrate medical imaging into EHR with a more accurate prediction and decision [27]. Such models, however, encounter the problem of supporting the complexity and heterogeneity of EHR data, particularly when also the data originate in more than one place. The success of multimodal AI implementation in the healthcare environment can only be achieved when data formats are standardized and that data fusion methods are developed muscularly [21].

Cross-modal integration or the ability to query visual and text data of other sources is a severe difficulty with healthcare AI. Considering that medical imaging would obtain EHR data, advanced methods should be created to handle the various types and forms of data [28]. One significant challenge will be associated with the ability to make sure that this AI model will be able to consolidate these multifaceted sources of data into suggesting generic accessibility predictions without drowning them in noise or inaccuracies [9]. In addition, in the multimodal AI systems, patient privacy and regulatory standards require the privacy of patient data. Nevertheless, the possible benefit of cross-modal integration in healthcare is enormous. This means that with the combination of medical imaging and EHR data, more precise and tailored treatment suggestions of AI systems are enabled that eventually results in a reduction in healthcare costs and patient outcome improvement, in the end resulting in better patient outcomes in general [29]. Subsequent research in cross-modal AI applications in healthcare must focus on advancing the rigor and generalizability of such systems and make sure that they can be generalized to different clinical environments [12].

### MAS in healthcare

MAS are a promising approach to enhancing clinical decision-making by leveraging the expertise of multiple autonomous agents. MAS are one of the promising solutions to the improvement of clinical decision-making when the skills of several agencies are used. These are systems wherein multiple AI models, each with a specific area of specializing in solving one of the healthcare issues, can interact to cooperate, communicate, and even interact to assist in solving the complex healthcare issues [30]. As an illustration, in a clinical decision support system, the first agent is the medical image assessor, the second agent is the patient history data assessor, and the third agent produces a report upon the given analysis. Such interplay of these agents can be used to increase the accuracy of diagnosis and positively affect treatment outcomes due to leveraged optimization of decision-making. Compared to traditional clinical workflows, MAS have been found to significantly increase a workflow in a diverse, complex setting with a high degree of simultaneity in completed tasks [31]. For example, MAS have previously been used to enhance diagnostic accuracy in pathology and radiology via the coordination of agents for image analysis, report generation, and clinical validation. In addition, these systems can optimize hospital operations by automating office tasks such as patient scheduling and resource allocation [32].

In medical imaging, segmentation is one of the most critical tasks, as it involves identifying and delineating regions of interest (ROIs) within an image. Automatic segmentation of anatomical structures in medical images has been automated via MAS where different agents ensemble to segment different anatomical structures [33]. For instance, one of the agents might deal with the segmentation of the brain in MRI, the other with the segmentation of tumors in CT, and so on. Through the distribution of workload among multiple agents, these systems show an ability to improve the efficiency and accuracy relative to the task, which can be tedious and vulnerable to human error in some cases. Medical image segmentation has been shown to be able to be solved better using MAS instead of single-agent approaches. The diversity of expertise across agents provides these systems a robust and accurate ability to segment [32].

Recent quantitative studies indicate that collaborative multiagent frameworks can outperform standard single-agent baselines in complex environments. For example, when using multiagent reinforcement learning to repeatedly optimize 3-dimensional medical image segmentation, the improvement in the Dice similarity coefficient (DSC), averaging a 3% increase in each instance (e.g., 85.56 to 88.53 on brain tumor data), was obtained over traditional single-agent convolutional networks [34]. Equally, cooperative multiagent deep reinforcement learning models on COVID-19 CT image segmentation had a high precision of 97.12% and high Dice scores (80.81%), indicating that representation of the segmentation task among specialized agents is effective in mitigating the oversegmentation and ambiguous boundaries addressed well, as compared to monolithic models [35]. Although interoperability and data consistency are still an issue in work, the heterogeneity of expertise targeted among interacting agents can offer these systems a considerably strong solution to medical image segmentation [31].

The future of MAS in healthcare is bright, and it can be applied in many spheres, such as diagnosis, treatment planning, and hospital management [36]. However, to make these systems applicable in the wide-ranging clinical practice, some obstacles should be surmounted. The capacity of MAS to operate in real time and especially speedy environments is a major challenge, particularly in clinical settings that are very time-related in nature [37]. In addition, the issues of data privacy, agent accountability, and system transparency must also be resolved for MAS to become something safe and ethical to manage in healthcare. However, MAS hold tremendous possibilities in improving the delivery of healthcare [38]. As AI technologies continue to get better, MAS are bound to play an instrumental role in automating and optimizing the clinical workflow that will lead to a higher level of efficiency and individuality of the care [32].

### Limitations of SLRs

The SLRs we have reviewed, focusing on AI and medical imaging, reveal several limitations specific to the current body of research. Those constraints are closely intertwined along with the character of the emerging AI technologies in healthcare and the existing challenges in integrating multimodal data, model architecture, and clinical realities. Failing to ensure the standardization of the study methods is by all means one of the largest shortcomings found in the reviewed SLRs. Considering the case of DL applications in medical image analysis, there are differences in the types of neural networks taken into account, image preprocessing methods, and measures of evaluation that make the direct comparison of the results practically impossible. The definition of success used in various studies (e.g., diagnostic accuracy, sensitivity, and specificity) and the various modalities of imaging investigated (e.g., CT scan, MRI, and x-ray) pose varying obstacles that must be met in various forms, and the comparison between various studies is therefore more challenging to make [14]. Definitive conclusions could not be made due to the failure to identify an explicit outline of standardization of the methodologies of studying that have been incorporated in the studies. As an example, during the medical image segmentation, though they execute the same procedure, one AI model is not tested according to an identical protocol alongside another model; thus, the results cannot be directly compared to each other directly [32].

Another limitation is the underrepresentation of real-world clinical data in the studies included in the reviews. In the majority of cases, the SLRs are focused on the results of the experiments conducted in controlled conditions on datasets (e.g., The Cancer Genome Atlas [TCGA] or Medical Information Mart for Intensive Care-Chest X-ray [MIMIC-CXR]), which can be extremely curated, though it is very uncommon that they are rich in variability encountered in the reality of a clinical setting of the experiment environment itself [20]. This constraint means that AI models may be highly performative in such dataset; however, the real applicability of their words to reality is not determinable. Currently, the research on the multicenter or multidevice modeling is limited and thus limits the applicability of AI models to any clinical environment with different imaging devices, protocols, and patient demographics in general [39]. One of the most visible instances of such is the review of image harmonization methods where the challenge of standardization of imaging data across centers and devices becomes highly simplified, and studies reviewed do not have a commentary on how models might address real-world issues of multiple centers/clinics [21].

Finally, the deficit of transparency and ethical consideration is another recurring problem throughout the analyzed SLRs. Nevertheless, the information regarding what we do with the bias of the data (e.g., demographic imbalance and clinical disparity) is lacking in many studies [18]. Also, when considering the technical performance of AI models, SLRs are likely to leave ethical concerns that accompany the use of an AI model in clinical practice. The reviews casually accepted patient privacy, model accountability, and explainable AI (XAI) decisions, which are pivotal to clinical adoption of AI tools (not always covered canonically in this group of papers) [32]. This lapse in the coverage of the ethical and social consequences of applying AI in medical imaging can delay the realization of the findings of research into clinical practice.

## Methodology

### Search strategy

A comprehensive and systematic search strategy is fundamental for ensuring the inclusion of relevant and high-quality literature. To broaden and widen the scope of the research that has been reviewed around pathology image analysis, DL, MAS, and LLMs, multiple reputable academic databases were chosen for this review [40]. To cover the medical imaging and AI area, the primary databases selected for use are PubMed, IEEE Xplore, Scopus, and arXiv, which include peer-reviewed journal articles, conference proceedings, and preprints.

Although the main search strategy was confined to digital pathology and WSI, additional medical imaging terms (e.g., “medical report” and “multimodal LLM” was important) were not strictly filtered to pathology in some query combinations. This was needed to be able to acquire very transferable underlying models and MAS in related areas such as radiology, which offer very important methodological background to the emergent pathology processes.

The search queries were carefully constructed to balance comprehensiveness and specificity. Key search terms and phrases were combined using Boolean operators (AND, OR) to capture literature spanning the intersection of fields. Representative keywords included the following:

• “Pathology image analysis”
• “Whole-slide imaging”
• “Deep learning”
• “Convolutional neural networks”
• “Transformer models”
• “Vision-language models”
• “Large language models”
• “Multi-agent systems”
• “Medical report generation”
• “Image-text generation”

**Figure 1.**
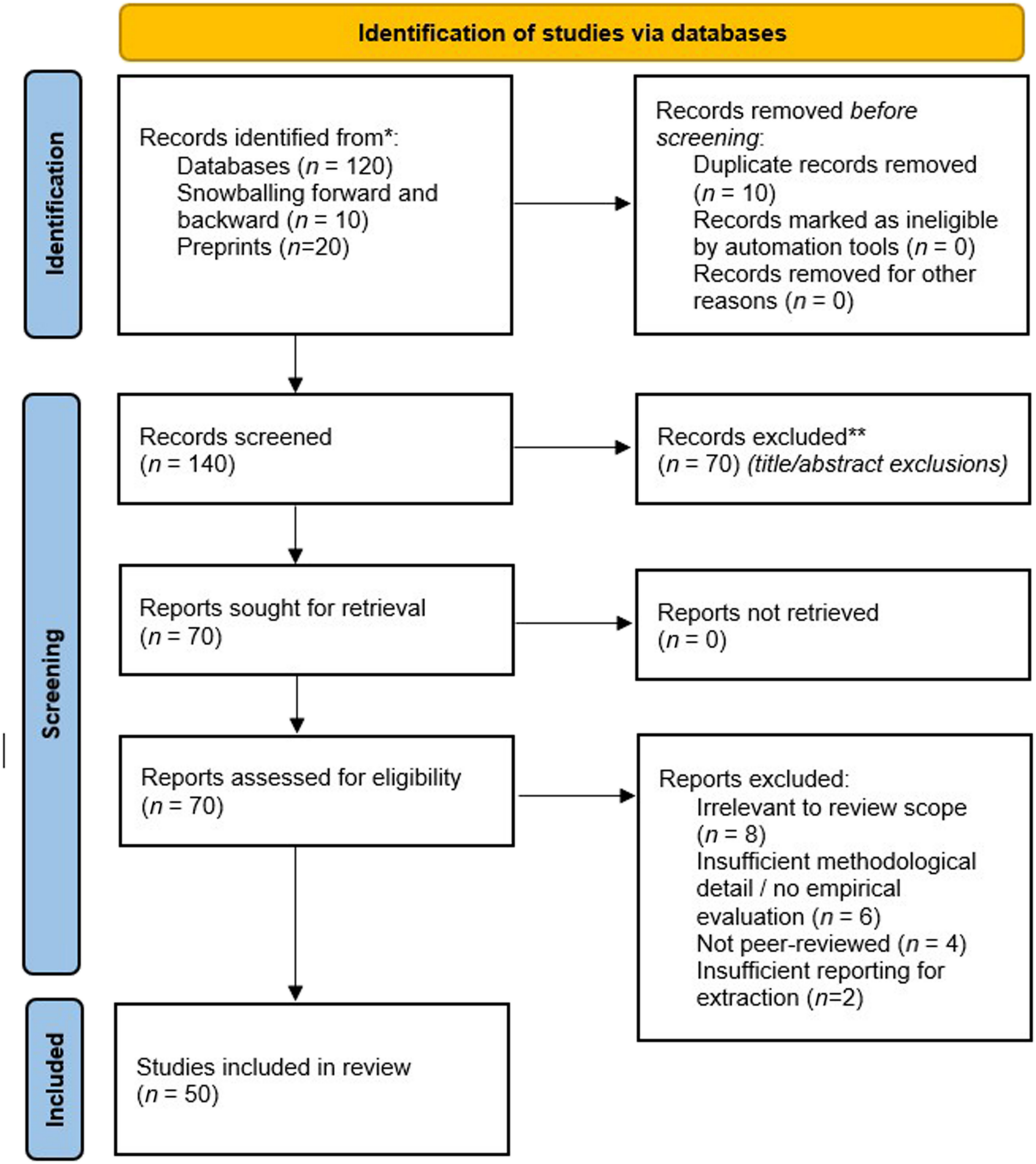
PRISMA (Preferred Reporting Items for Systematic reviews and Meta-Analyses) study selection process flow diagram.

Searches were conducted iteratively, refining terms based on preliminary results to ensure relevant papers were not overlooked. Figure 1 shows the complete process of article selection.

### Database search strategy and search strings (April to May 2025)

To ensure reproducibility, we executed database-specific search strings across 4 sources: PubMed, Scopus, IEEE Xplore, and arXiv. Searches were conducted between April and May 2025. The strategy targeted 3 intersecting concepts: (a) digital pathology/WSI, (b) DL for pathology image analysis, and (c) VLMs/LLMs and multiagent frameworks for image-to-text generation and decision support. Where supported, we searched titles/abstracts/keywords, applied an English-language filter, and restricted publication years to 2015 to 2025.

#### PubMed (April to May 2025)

PubMed was queried using Title/Abstract fields (and MeSH where relevant) to capture biomedical terminology. The following query was executed with filters: English; publication dates 2015 January 1 to 2025 December 31.

**PubMed query (Title/Abstract) (*n* = 34):**
((“digital pathology”[Title/Abstract] OR “whole slide imaging”[Title/Abstract] OR WSI[Title/Abstract] OR histopatholog*[Title/Abstract] OR “computational pathology”[Title/Abstract])AND(“deep learning”[Title/Abstract] OR “convolutional neural network”[Title/Abstract] OR CNN[Title/Abstract] OR transformer[Title/Abstract] OR “vision transformer”[Title/Abstract] OR ViT[Title/Abstract] OR “multiple instance learning”[Title/Abstract] OR MIL[Title/Abstract] OR “foundation model”[Title/Abstract]) AND (“vision-language”[Title/Abstract] OR “vision language”[Title/Abstract] OR multimodal[Title/Abstract] OR “image-text”[Title/Abstract] OR “image to text”[Title/Abstract] OR caption*[Title/Abstract] OR “report generation”[Title/Abstract] OR “large language model”[Title/Abstract] OR LLM[Title/Abstract] OR “medical report”[Title/Abstract] OR “pathology report”[Title/Abstract] OR “multi-agent”[Title/Abstract] OR “multi agent”[Title/Abstract] OR agentic[Title/Abstract]))


#### Scopus (April to May 2025)

Scopus was searched using TITLE-ABS-KEY to improve coverage of computer science venues and interdisciplinary publications. Filters were applied for the following: English; years 2015 to 2025; and document types (article, conference paper, and review excluded at screening stage).

**Scopus query (TITLE-ABS-KEY) (*n* = 38):**
 TITLE-ABS-KEY((“digital pathology” OR “whole slide imaging” OR WSI OR histopatholog* OR “computational pathology”) AND (“deep learning” OR “convolutional neural network*” OR CNN OR transformer* OR “vision transformer” OR ViT OR “multiple instance learning” OR MIL OR “self-supervised” OR “foundation model*”) AND (“vision-language” OR “vision language” OR multimodal OR “image-text” OR “image to text” OR caption* OR “report generation” OR “medical report*” OR “pathology report*” OR “large language model*” OR LLM OR “multimodal large language model*” OR MLLM OR “multi-agent” OR “multi agent” OR agentic OR “collaborative agent*”))AND (LIMIT-TO(LANGUAGE, “English”))AND (PUBYEAR > 2014 AND PUBYEAR < 2026)


#### IEEE Xplore (April to May 2025)

IEEE Xplore was searched to capture engineering and computing studies (e.g., MIL, transformers, multimodal learning, and agent-based frameworks) often not indexed uniformly in biomedical databases. Searches were applied to Metadata (Title, Abstract, and Index Terms) with filters: English; years 2015 to 2025.

**IEEE Xplore query (Metadata) (*n* = 26):**
((“digital pathology” OR “whole slide imaging” OR WSI OR histopathology OR “computational pathology”) AND (“deep learning” OR CNN OR “convolutional neural network” OR transformer OR “vision transformer” OR ViT OR “multiple instance learning” OR MIL OR “foundation model” OR “self-supervised”) AND (“vision-language” OR “image-text” OR multimodal OR “report generation” OR “medical report” OR “large language model” OR LLM OR “multimodal LLM” OR MLLM OR “multi-agent” OR agentic))


#### arXiv (April to May 2025)

arXiv was searched to capture rapidly emerging work in multimodal foundation models and agentic systems relevant to digital pathology. Searches were executed over title and abstract terms. We limited results to 2015 to 2025 and English-language manuscripts.

**arXiv query (title/abstract keywords) (*n* = 22):**
(“digital pathology” OR “whole slide imaging” OR WSI OR histopathology OR “computational pathology”) AND (“deep learning” OR CNN OR transformer OR “vision transformer” OR ViT OR “multiple instance learning” OR MIL OR “foundation model” OR “self-supervised”) AND (“vision-language” OR “image-text” OR multimodal OR “report generation” OR captioning OR “large language model” OR LLM OR “multimodal LLM” OR MLLM OR “multi-agent” OR agentic)


#### Search refinement and auditability

Searches were iteratively refined to reduce false positives while maintaining recall, primarily by (a) requiring explicit pathology/WSI terms, (b) retaining both classical DL (CNN/MIL) and transformer/foundation-model terms, and (c) including VLM, LLM, and multiagent keywords to capture integrated image-to-text and agentic frameworks. The final strings above are reported verbatim for auditability and reproducibility.

### Inclusion and exclusion criteria

To maintain focus and relevance, specific inclusion and exclusion criteria were defined prior to the selection of studies:

#### Inclus

• Studies published in English between 2015 and 2025 to capture recent advances.
• Peer-reviewed journal articles, conference papers, and preprints with significant technical contributions.
• Research focused on pathology or closely related medical imaging fields (e.g., radiology) involving DL or multiagent frameworks.
• Studies addressing image analysis, report generation, or the integration of visual and textual data.
• Works discussing datasets, evaluation metrics, or clinical applications relevant to pathology image–text tasks.
• Preprints were included to capture the latest research in AI for pathology, addressing uncertainty through a quality assessment that considered peer review status and methodological rigor, prioritizing later peer-reviewed studies.
• Nonpathology studies were included only if their methodologies, such as AI models or MAS, were directly applicable to pathology image analysis, ensuring relevance to the scope of the review.

#### Exclusion criteria

• Papers unrelated to medical imaging or pathology.
• Studies focusing solely on traditional image processing without AI or machine learning methods.
• Articles lacking sufficient methodological detail or empirical evaluation.
• Review papers, opinion pieces, or editorials (though references within these were screened for additional sources).

### Study selection process

The selection process followed a rigorous screening protocol to ensure only the most pertinent studies were included. Initially, all search results were imported into a reference management tool, where duplicates were removed. Two rounds of screening were conducted:

The selection process followed a rigorous screening protocol to ensure only the most pertinent studies were included. The steps of the selection process were as follows:

• Title and abstract screening: Each paper was assessed for relevance based on its title and abstract. Studies that were clearly outside the scope or did not meet the inclusion criteria were excluded.
• Full-text review: The remaining papers underwent a detailed full-text review to confirm their alignment with the research objectives and methodological standards.
• Reviewer logistics: The screening process involved 2 independent reviewers who assessed the studies based on predefined inclusion/exclusion criteria.

To ensure the robustness of the full-text screening phase, interrater reliability was quantitatively assessed. The agreement between the 2 independent reviewers was substantial, yielding a Cohen’s kappa of 0.72 (87.1% observed agreement). The 9 remaining conflicts were resolved by a third reviewer through discussion and consensus, resulting in the final corpus of 50 studies.

### Data extraction and synthesis

For each included study, a structured data extraction form was developed to capture essential information systematically. Key data points included

• Publication details: authors, year, and venue
• Study objectives and scope
• Technical methodologies: model architectures (CNN, transformer, VLM, and LLM), MAS design, and training strategies
• Datasets used: type, size, and annotation details
• Evaluation metrics and results: accuracy, F1-score, BLEU score for text generation, and clinical validation if available
• Challenges and limitations noted by authors
• Clinical applicability and integration aspects

The extracted data were synthesized qualitatively to identify trends, common approaches, and gaps. Quantitative meta-analysis was considered but limited due to heterogeneity in tasks, datasets, and evaluation protocols.

### Quality assessment of included studies

Quality assessment was conducted to evaluate the robustness and reliability of each study. Criteria included the following:

• Methodological rigor: clear description of algorithms, data preprocessing, and experimental setup;
• Reproducibility: availability of code, datasets, or detailed protocols;
• Evaluation thoroughness: use of appropriate metrics, baseline comparisons, and validation on multiple datasets;
• Clinical relevance: alignment with real-world pathology challenges, inclusion of domain knowledge, or clinical expert validation;
• Transparency about limitations and biases.

Studies scoring below a predetermined threshold (0 to 3) on these quality assessment criteria were carefully evaluated before making a final decision on their inclusion. Any study that had serious methodological limitations, including ambiguous experimental design, inadequate validation, improper description of the data, or no clinical relevance, was usually dismissed during the main synthesis to maintain a high level of rigor of the review. Nonetheless, when the research presented a new interpretation or inquiry of a new subject, even in a minor manner, it was put on the table of discussion with reservations. In these circumstances, though, the constraints and potential biases were clearly mentioned and the results were continuously interpreted in such a way that there is no overgeneralization of the findings. This moderate stance justifies the scientific soundness of the review and also formulates and brings to actual realization the thought experiment of what a comprehensive review may appear like, having a moderate perspective of potential directions toward success and simultaneous compensatory and joint failures of the extant literature.

To operationalize this quality assessment, each of the 5 criteria (clarity, reproducibility, evaluation rigor, clinical relevance, and transparency) was evaluated using a 4-point ordinal scale: 0 (poor), 1 (moderate), 2 (good), and 3 (excellent). The overall unweighted average of all these 5 dimensions resulted in the final quality score of every study. According to this rubric, the final set of 50 studies that were used had a very high overall methodological standard. The number of the studies that were categorized as “excellent” (average score of 2.5 to 3.0, with 9 studies scoring 3.0 on the point scale) is 43. The other 7 articles were rated as good (average score of 2.0 to 2.4). On the last point, it was important to note that none in the final corpus were classified in the moderate (1) or poor (0) category, thus attesting to the fact that all the included papers comfortably met this minimum quality requirement to be included.

The dataset used in this study, including all papers reviewed, the data extraction table, and quality assessment results, is available in the repository with DOI: https://doi.org/10.5281/zenodo.18674518. The repository ensures the transparency and reproducibility of the SLR process.

### Deduplication procedure (Bibcitation)

All records retrieved from the database searches and forward/backward snowballing were imported into Bibcitation for reference management and deduplication. Duplicates were first detected automatically using exact identifier matching (DOI and PMID/arXiv ID where available). A secondary pass was then performed using bibliographic similarity rules (exact/near-exact title matching, combined with year and first-author checks) to capture records with missing identifiers or minor formatting differences. Finally, all flagged pairs were manually verified to prevent erroneous removals (e.g., conference and journal extensions treated as distinct records when substantially different). This process removed 10 duplicate records, yielding 140 unique records for title/abstract screening.

## Analysis

### Overview of included studies

This section provides a summary of the key characteristics and scope of the studies included in this SLR. In the selected studies, authors focus the research on applying DL and VLMs to pathology image analysis, as well as the use of MAS to generate clinical reports of the analysis. They collectively represent the latest developments, datasets, methodologies, and challenges in the domain at large.

#### Study distribution and research focus

The majority of the reviewed studies fall within 3 main thematic areas:

• DL for pathology image analysis: Several studies have developed or refined DL architectures tailored to the unique challenges of digital pathology images. The segmentation, detection, and classification of histopathological structures with CNNs, transformer-based models, or hybrid frameworks are the main focus of these works. For instance, Janowczyk and Madabhushi’s extensive research on CNN applications in digital pathology, Kosaraju et al.’s HipoMap, a slide-based representation framework, and Neidlinger et al.’s Efficient Approach for Guided Local Examination (EAGLE) framework with a focus on efficient tile selection and feature extraction in WSIs [41–43].
• VLMs for image–text pair generation and report automation: Studies like those by Sun et al. [44] with PathGen-1.6M and Shi et al. [45] with ViLa-MIL highlight the integration of vision and language models to generate clinically relevant textual descriptions and diagnostic reports from pathology images. Other works include Ding et al.’s TITAN foundation model for multimodal learning including WSIs and pathology reports at the scale that achieves strong generalization across multiple tasks without fine-tuning [46].
• MAS and collaborative frameworks: Emerging research illustrates the promise of multiagent approaches where specialized AI agents handle distinct subtasks, such as feature extraction, text generation, and validation. Systems like Large language model Extractor for Abdominal Vision Supervision (LEAVS) and Multi-agent Collaboration-based Compositional Diffusion (MCCD) demonstrate how collaborative agents improve annotation quality and generate synthetic training data, respectively, thereby enhancing the overall system performance and clinical utility [47,48].

#### Data sources and datasets

The studies reviewed exploit diverse categories of both publicly and privately available datasets, including dedicated datasets in the form of TCGA and giant datasets of recently created pairs between pathology images and their text typically (PathGen-1.6M and Quilt-1M) [44,49]. These datasets can be used to train and test models on various types of cancer, image type, and during the clinical scenario, which enables the creation of versatile and general AI tools.

#### Methodological approaches

Approaches range in both directions between fully supervised CNN models and weakly supervised learning models, e.g., clustering-constrained attention multiple-instance learning (CLAM), and more sophisticated transformer and self-supervised learning models. In order to address the weakness of the quantity of labeled data, researchers use many techniques, typically data augmentation, synthetic image creation, and certain active learning (AL) methods. Moreover, domain-specific knowledge integration is provided, in order to make generated text correspond to clinical terminology and guidelines.

### Evaluation metrics and performance

Performance evaluation consists of image-specific (e.g., F-score, accuracy, and area under the receiver operating characteristic [AUROC]) and NLG (e.g., BLEU and clinical relevance measures) metrics used to evaluate performance across both modalities of image depiction and NLG [50]. Certain frameworks are demonstrated to be able to process at real time or near real time, which is essential to clinical deployment, and most studies have shown their capabilities to be better than handcrafted feature methods and older AI models.

Conventionally, the AI medical reports were evaluated against the usual measures of NLG such as BLEU, ROUGE, and METEOR. Though these are convenient in measuring syntactic fluency and overlaps between the text and reference report against a ground truth, the measures are basically a sham in the medical profession. A genuine report may score a high BLEU score in a situation where the resultant generated text replicates boilerplate text; thus, the generated text does not contain a very crucial diagnostic specifier that results in a calamitous clinical error [50].

#### Evaluation metrics and operational definitions

In an attempt at providing formal measures of comparative heterogeneous studies in different multimodal and multiagent constructs, the use of evaluation measures and operational terms used when conducting this review must be clearly defined. Every specific metric is preconditioned by the type of the underlying task:

• Classification and diagnostic tasks: The area under the receiver operating character parallel curve (AUC) is used to classify the performance in most cases. The main benefit of AUC is that it quantifies the power of a model to differentiate between classes (e.g., benign vs. malignant tissue) at any level of classification; thus, it is much more suitable and resilient to apply to inherently imbalanced datasets such as those in pathology.
• Segmentation and spatial grounding tasks: Tasks that must localize anatomical structures are based on the DSC and Intersection over Union (IoU). These measures determine the spatial overlap of the AI-predicted boundaries and the ground-truth pathologist annotation, needed to assess tumor delineation, as well as functional visual grounding of VLMs.
• Syntactic text generation: Traditional NLG tasks use metrics like BLEU, ROUGE, and METEOR to measure n-gram (word sequence) overlap between AI-generated text and reference reports. While appropriate for assessing the syntactic fluency of a generated report, they cannot measure factual medical accuracy.
• Semantic report generation: To evaluate true medical utility, studies increasingly rely on clinical correctness and clinical efficacy (CE) metrics. These evaluate the factual accuracy of the text (e.g., successfully predicting diagnostic tags and omitting contradictory hallucinations) rather than simple word-matching.

Furthermore, several repeated qualitative claims extracted from the reviewed literature require strict operational definitions:

• Acceptance rate: This review operationally defines acceptance rate as the percentage of AI-generated reports or diagnostic recommendations successfully utilized in a clinical workflow by an expert pathologist with no or only slight grammatical changes. It is based on this definition because the human-in-the-loop reader studies were removed directly out of the review process.
• Clinically accurate: An AI output is considered to be clinically accurate when it can detect the main diagnostic characteristics (i.e., tumor grade and histological subtype) and provide no conflicting data or omit the most important nominal (e.g., the word no translated before a modifier). This is confirmed by grading schemes of clinicians in a structured manner and not by automated text measurements.

#### Balancing NLG with clinical correctness and clinician grading schemes

To bridge the gap between textual similarity and medical accuracy, recent frameworks increasingly balance conventional NLG metrics with rigorous CE metrics and structured clinician grading schemes. For example, the analysis of the PathAlign framework is done by a special pathologist-based grading scheme. It does not rely on simple text comparison, rather it evaluates whether there are any clinical significant errors or omissions present or absent predetermined issues on an individual basis [51]. Pathologists carry out a manual review of the generated text in their reader studies to verify that the generated text is able to be used to reach the diagnostic conclusion and prioritize the workflow without hallucinatory finding.

Similarly, models like the patient-level multiorgan pathology report generation (PMPRG) framework emphasize the necessity of tracking clinical correctness in complex, inhomogeneous multiorgan reports. PMPRG uses targeted CE measures to assess the ability of the formulated reports to make the right predictions and include predefined diagnostic “tags” to supplement standard NLG measures such as METEOR [52]. Through assessing the accuracy of tag classification, the researchers can measure quantitatively whether the generated text is effective in capturing the key organ-specific diagnostic features as opposed to merely generating generic and fluent text.

#### Grounding metrics and spatial interpretability

Moreover, assessing the factuality of a report would be accomplished by demonstrating that the textual claims of the AI are based on the right visual evidence, as opposed to being hallucinated by the statistical priors of the language model. This has seen the incorporation of grounding measures in the evaluation of reports. The grounded report generation process necessitates the model to match particular textual assertions with accurate X/Y coordinates or ROIs, on the entire slide image.

Measurement of this spatial correspondence means comparing model predicted attention maps or multiscale regional features with pathologist-labeled ROI. Grounding metrics ensure an audit trail, hence requiring models to explain the origin of their generated text with reference to its visible basis of evidence. This makes the form of the pathology report produced not only syntactically fluent and clinically sound, but also correctly pegged to the underlying morphological reality of the tissue sample.

### The weak supervision challenge and MIL evolution in WSI analysis

Since WSIs are gigapixels by definition, it is virtually impossible to perform exhaustive pixel-wise annotation of such images by highly trained, expert pathologists. This means that only one slide-level diagnostic label (i.e., malignant or benign) can be used to train models even though the morphological evidence involved in the actual diagnosis may be a minute fraction of the scanned tissue. To address this, multiple instance learning (MIL) has been discovered to be quite useful with the field with a WSI being viewed as an instance of a bag of smaller, unlabeled image patches or instances [53].

MIL architectural developments have followed a series of different paradigms to reflect more accurately complex tissue morphology through weak supervision. In early techniques, simple pooling (max or mean pooling) was used, and this was not capable of isolating individual sections of diagnostic interest in background tissue. This shortcoming resulted in the accomplishment of attention-based MIL whose dynamically ascribed learnable weight values to the patches allows the model to visually target ROIs. In order to increase the unrestrictedness of discerning the extents of the spatial correlations amid the tissue structures of further areas, the field moved back to transformer-based models with TransMIL being the most eminent. More recently, to further justify the large-scale sequences of WSI patches with further computational power, scholars modified state space models that formulate infrastructures like Mamba-MIL. Meanwhile, other advanced clustering-based models have been suggested, such as MiCo, which enhances the feature representations through the contrastive and pseudo-labeling learning. This history of the simplest kind of pooling, to Mamba-MIL, to MiCo is important to comprehending why the approaches to multimodal models are effective nowadays to ground their clinical text generation at a larger scale, and unsupervised visual space [54].

The evolution of attention-based and transformer-driven MIL is not merely an endpoint for slide-level classification; it serves as the critical visual engine for the advanced multimodal and multiagent frameworks discussed in subsequent sections. For instance, modern MIL architectures produce attention weights and spatial hierarchies that are automatically used as natural heatmaps to determine the prioritization of ROI in multiagent pipelines. PathFinder [55] and SlideSeek [56] systems are based on these localized signals to steer their Navigation and Explorer Agents to a diagnostically rich area in the gigapixel expanse. In addition, structured patch aggregation is a fundamental requirement of VLMs. Such multiscale visual embeddings are used in frameworks such as PathAlign [57], which facilitates region-aware grounding, in which the generated clinical text is aligned with particular cellular morphologies. Finally, the combination of such complex representations of MIL works as one of the major protective measures against diagnostic hallucinations. These architectures achieve strict control over the text-generation process through high-attention, clinically relevant tissue patches instead of the unfiltered WSI background by imposing downstream language models with other unsupported clinical claims [58,59].

### DL approaches for pathology image analysis

DL has emerged as a key technology in digital pathology, which has provided revolutionary advances in image processing via automated characteristic study of raw information directly at the information stage [60]. Examination of pathology images presents very special problems of very large-resolution WSIs, complex tissue structure, and the extreme variability of the staining procedure and imaging equipment used [61]. The state of the art of working with the DL approaches used in the analysis of pathology images is described, including the review of the model architectures, data strategies, and clinical application as outlined in the literature.

#### CNN-based architectures and segmentation frameworks

Janowczyk and Madabhushi [41] had first performed early foundational work demonstrating the versatility of CNNs in digital pathology that was widely featured in their work. They tested CNN in working with several tasks: nuclei segmentation, epithelium and tubule segmentation, lymphocytes and mitosis, and cancer classification. Their strategy involved Caffe DL framework, high F score (maximum of 0.90 in lymphocyte detection), and aspects of the approach like choice of magnification and label quality that will be suitable in clinical translation. In regard to a thorough assessment, CNN hegemony in pathology images was logical [41]. Huang et al. [62] augmenting CNN capabilities combine advanced segmentation U-Net with classification EfficientNetV2 with a novel algorithm of constructing a heatmap, grounded on data enhancement, ensemble learning, and attention mechanism into a new algorithm. It was revealed that the multicomponent framework only reached unbelievable accuracy (precision 92.86%, recall 86.67%) on the TCGA task of identifying cancer lesions that proves the importance of sophisticated fusion of models to enhance both the performance and scalability of analyzing pathological images [62].

To overcome the limitation of data scarcity and annotation, Hossain et al. [63] added synthetic image generation using a cycle-consistent generative adversarial network (GAN) to train using nuclei segmentation. The accuracy of segmentation has increased the DSC of 0.984; also, the segmentation under models with synthetic images against models with original images is elevated (DSC 0.805). The scarcity of the annotated pathology images is characterized by the essential role of the synthetic data creation and the advanced pipeline of preprocessing marked as the key issue to the future of the research field [63]. In the same way, Zhang et al. [64] reported the application of CNN-based histopathological image analysis to predict the risk of further progression of oral leukoplakia, and the DL models could distinguish morphological patterns that are specific to the risk of cancer occurrence [64].

#### Slide-level and whole-slide image representations

While patch-wise CNN analysis is common due to computational constraints, slide-level diagnosis demands aggregation across large WSIs. Kosaraju et al. [42] introduced HipoMap, a slide-based framework converting WSIs into structured representations compatible with CNNs. HipoMap is a slide-based framework introduced by Kosaraju et al. to convert WSIs into a structured representation for compatibility with CNNs. HipoMap was shown to outperform existing methods for lung cancer classification (AUC 0.96) and survival prediction (c-index 0.787) and presented a flexible, task-agnostic, slide-level pathology analysis that meets the needs of the clinical participant [42]. Slide representation learning methods are increasingly being explored in an unsupervised way. PANTHER (Song et al. [65]), is a Gaussian mixture model-based approach to derive morphological prototypes in the space of WSI patches, resulting in expressive, task-agnostic slide embeddings. On 13 datasets, their method matched or surpassed the performance of supervised MIL approaches and provided improved interpretability through prototype analysis, which is a major advantage toward clinical adoption [65]. In particular, Ding et al. [46] extended multimodal integration with the development of TITAN, a large-scale foundation model pretrained on over 335,000 WSIs that have been provided with associated pathology reports and synthetic captions. Zero-shot and few-shot generalization with this self-supervised learning and vision-language alignment to diverse clinical tasks such as retrieval of rare cancer from whole slide images, and generation of pathological reports, demonstrate a possible new frontier of DL models incorporating both image and text modalities to provide versatile clinical support [46].

#### Efficiency and scalability in high-resolution image analysis

Processing gigapixel WSIs remains a computational bottleneck. EAGLE, an efficient DL framework for emulating pathologist workflows based on 2 foundation models, CHIEF for tile selection and Virchow2 for feature extraction, is presented by Neidlinger et al. [43]. It is shown that EAGLE reduced the computational time by over 99% across 31 cancer-related tasks to near-real-time processing speed (2.27 s per slide) while achieving performance superior to the aforementioned leading models. EAGLE's clinical application does not require high-performance computing resources [43]. Meirelles et al. [66] designed an AL framework that acquires data with diversity-aware data acquisition and network auto-reduction techniques to reduce expert annotation time without sacrificing model accuracy. Their approach is then applied to tumor-infiltrating lymphocyte classification and achieves superior predictive performance at up to 4.3× reduction in execution time for AL, while simultaneously addressing one of the key challenges in applying DL to pathology: high annotation costs [66].

#### Addressing variability and robustness: Data augmentation and domain adaptation

The heterogeneity of pathology images, influenced by staining differences, scanning protocols, and patient populations, poses significant challenges to model generalizability. The literature uses extensive data augmentation as a strategy to overcome the issues of overfitting and robustness [41,62] as well as synthetic data generation [63]. Similarly, Lu et al. proposed CLAM, a weakly supervised attention-based MIL model, which makes use of slide-level labels to limit the intensive annotation of the patches and guarantee model versatility in cohort and/or imaging contexts. Such an interpretable attention installation provided by CLAM focuses on diagnostically significant parts that are more reliable in the eyes of the clinician and more intelligible to the model [67]. A number of studies indicate that the incorporation of domain-specific knowledge can be useful in order to leverage clinical transposability. Recently, Zhang et al. [64] demonstrated that risk stratification models based on CNN can be improved by means of integrating the observed histological patterns with the clinical outcomes associated with them in patients with breast cancer of the second and third stages of the disease. Huang et al. [62] also enabled them to capitalize on pathological expertise and influence both attention mechanisms and ensemble strategy that contributed to interpretability and could acquire precise information on the topic. Having incorporated this, the difference between the results provided by the AI and the real clinical decision-making processes is narrowed, and the results provided by the generation become valuable and practical.

#### Comparative analysis and limitations

Across the reviewed DL architectures, a clear evolutionary trade-off emerges between annotation dependency and clinical interpretability. Wholly trained CNN models are still the best at handling localized accuracy challenges, e.g., nuclei segmentation, but are fundamentally constrained by the prohibitive cost of annotating pixels pointwise [42,65]. To avoid this bottleneck, the domain has a common architectural jump to weakly supervised MIL (e.g., CLAM) and self-supervised foundation models (e.g., TITAN and PANTHER). Nonetheless, this paradigm comes with a nonconsecutive design bottleneck: as models are made less supervisory on the pixels and more move to supervisory on the slide, the problem of spatial interpretability to clinicians increases exponentially. In turn, hybrid frameworks, e.g., hybrid EAGLE [43] and AL pipelines [66], that sensibly trade off the high-level contextual understanding of the foundation models with high-efficiency tile selection seem to be the most viable near-term clinical solutions in that they can both be fast and interpretable.

### VLMs and LLMs in medical report generation and image–text pair generation

The integration of VLMs and LLMs has brought a paradigm shift in the medical image analysis and report generation. Integrating the capability to read and interpret intricate visual content and the capacity to write consistent and clinically useful text, these models are revolutionizing diagnostic processes in radiological, pathological, and other specialties relying heavily on imaging diagnostics, visual, and textual material [9]. This section discusses the current state of VLMs and LLMs in the medical fields; gives a comprehensive overview of the existing methodologies, datasets, architectures, and clinical applications; and outlines emerging issues.

#### LLMs in medical question answering and clinical dialogue

LLMs, initially designed for broad NLP tasks, have been adapted and fine-tuned to address the specialized requirements of medical question answering and dialogue systems [5]. This field includes models such as GPT-4, ChatGPT, LLaMA (Touvron et al. [68]), and PMC-LLaMA (Wu et al. [69]) and domain-tuned variants like MedPaLM (Singhal et al. [70]) and MedPaLM 2 (Singhal et al. [57]). For instance, MedPaLM 2 gains a 19% improvement in accuracy on the MedQA benchmark through fine-tuning and prompt engineering with more domain-specific knowledge [57]. Furthermore, Nanayakkara et al. [71] have shown that these LLMs can be used for ASR and transcription error correction in clinical conversations by using seq2seq approaches that use T5 and BERT architectures [71]. As such, this development caters to the practical need of accurately documenting clinical dialogue, a basis for other NLG tasks.

Despite impressive progress, the performance of general-purpose LLMs is often constrained by gaps in specialized medical knowledge and the inherent complexity of clinical reasoning. Duong and Solomon [72] found that the genetic question answering performance of ChatGPT was on par with human experts, but fell short in comparison to human experts, demonstrating the weakness of a pretrained model entirely devoid of domain-specific adaptation [72]. In response, researchers come up with systems such as ChatDoctor (Li et al. [73]) to augment LLaMA with self-contained information retrieval mechanisms, retrieving additional information from Wikipedia in real time in order to increase the accuracy and relevance of responses [73]. Clinical Camel (Toma et al. [74]) also uses dialog-based knowledge encoding with session memory and active knowledge base expansion to exceed GPT-3.5 on USMLE results and enable rich case management and generation of clinical documentation. An important trend is that hybrid systems enhance pretrained LLMs with external knowledge bases or retrieval modules to surmount the limitations of static training corpora. As examples of culturally and linguistically sensitive medical LLMs, Chinese LLMs include DoctorGLM (Xiong et al. [75]), Zhongjing (Yang et al. [76]), BenTsao (Yang et al. [77]), and Huatuo (Wang et al. [78]). Zhongjing improves complex dialogue and question handling using reinforcement learning with human feedback, and DoctorGLM provides precise symptom, diagnosis, and treatment guidance in Chinese by its prompt design and disease knowledge libraries [75,76].

Although originally intended as a more general clinic conversation, the active knowledge retrieval tools applied in these tools can be easily transferred to pathology. They could be reconfigured to interface with external genomic databases or histopathology grading systems (e.g., Nottingham grading of breast cancer) in real time as additional important contextual information to analyzing whole-slide images.

#### LLMs for clinical documentation and medical report generation

Automation of clinical documentation is a prime target for LLM applications, promising to reduce clinician workload and improve report standardization. According to Cascella et al., [79], ChatGPT can generate medical notes for intensive care unit patients with high accuracy in categorizing complex physiological parameters and LLM tends to be able to self-correct. Building on this work, Ali et al. [80] scaled this work to generate high-quality clinical letters across different scenarios with letters generated faster and with better patient satisfaction than in manual documentation. Given their larger parameter size and training data, the results reported by Waisberg et al. [81] and Abdelhady and Davis [82] demonstrate that GPT-4 can produce surgical discharge summaries, interpret medical images, and handle complex clinical trial documentation. GPT-4 was also efficacious compared to ChatGPT 3.5 when tasked with EHR inbox management, with admin and chronic disease management contexts favoring GPT-4 for hospital adoption.

A number of studies have been conducted in the field of radiology to examine how GPT can be used to process unstructured free-text reports and convert them into structured formats. Mallio et al. [83] investigate applications of GPT-3.5 and GPT-4 to Italian CT reports and report up to 75% word count reduction and improved clinical recall in GPT-4. Mallio et al. also examined the knowledge of GPT-4 regarding structured reporting, and established that in fact GPT-4 has a highly strong knowledge of structured reporting, and can generate large and detailed structured templates. Adams et al. [84] established a 100% success rate of automated translation of English radiology reports to structured x-ray JSON formats and a high success rate of 100% on German chest x-ray datasets, and promising multilingual, multitask promise of LLM. In distal radius fracture reporting, Bosbach et al. [85] experimented with GPT-3.5 and found high scores in grammar and style, certain problems in medical interpretation associated with realization, and that the area knowledge is necessary to be sensitive enough. Wang et al. [86] argue that the output of GPT-4 structured liver ultrasound reporting was more diagnostic and efficient compared to conventional free text reporting. Then, Jiang et al. [87] compared GPT-3.5 to GPT-4 concerning thyroid ultrasound reporting, where GPT-4 was observed to be more effective over GPT-3.5 in terms of nodule hostility, as well as in the consistency of the management recommendation.

Li et al. [88] suggested a novel pipeline, which is an interactive fusion that detects anatomical regions and prompts generated GPT-4 reports simultaneously on the first sight of a chest x-ray. According to this, their style was found to enhance anatomical and clinical detailization that is also another tendency in terms of more interactive and understandable LLM-driven monitoring systems. Additionally, Pan et al. [89] demonstrate that GPT-4 can produce Fast Healthcare Interoperability Resources (FHIR)-compliant structured radiology reports for multiple modalities, with high accuracy and internal consistency, ready to be incorporated into a standardized healthcare framework for data.

The effectiveness of these models to translate free-text radiology reports to structured forms gives a first-hand guide to computation pathology. A closely related NLP pipeline can be applied to histopathology to automatically identify standardized synoptic elements of report (including tumor margins, mitotic rate, and lymphovascular invasion [LVI]) in historically unstructured surgical pathology reports.

#### VLMs for medical report generation and VQA

VLMs extend LLMs by jointly processing images and text, enabling multimodal medical report generation and VQA. Some of the state-of-the-art VLMs in pathology include MedViLL (Moon et al. [90]), PubMedCLIP (Eslami et al. [91]), RepsNet (Tanwani et al. [92]), and BiomedCLIP (Zhang et al. [93]); their architectures were discussed and their performances were evaluated on datasets like MIMIC-CXR, Open-I, VQA-RAD, and SLAKE. In particular, MedViLL stacks a ResNet-50 visual encoder with a BERT (based) textual encoder that includes positional embeddings into a unified transformer architecture. Fine-tuned on smaller datasets and pretrained on nearly 90,000 image–report pairs, it achieves BLEU-4 scores around 0.06 and clinical label accuracy above 84%, generalizable to radiology report generation and question answering [90].

PubMedCLIP enhances contrastive learning on biomedical image–text pairs using ViT-B and transformer text encoders, achieving superior VQA accuracy when integrated with question-conditioned reasoning frameworks [91]. By combining ResNeXt-101 image encoding, BERT text embedding, and GPT-2 decoding and jointly learning via bidirectional contrastive and cross-entropy losses, RepsNet is shown to achieve competitive BLEU scores on medical image captioning [92]. Using VQGAN tokenization and transformer generations, UniXGen (Lee et al. [94]) innovatively generates both chest x-rays and radiology reports, trained on over 200,000 studies and representing a high-performance multimodal generative method. To improve VQA on various benchmarks, RAMM (Yuan et al. [95]) introduces retrieval augmented attention mechanisms that incorporate retrieved image text pairs from large biomedical corpora.

Contrastive X-ray Report Match (X-REM) by Jeong et al. [58] tackles the challenge of hallucinated information in radiology report retrieval and generation using Align the image and text representations Before Fusing (ALBEF)-based multimodal encoders and clinical label-informed ITM scoring, enhancing report relevance and semantic accuracy. To generate a radiology report that is grounded on high similarity retrieved impressions while simultaneously minimizing hallucination and improving clinical fidelity, CXR RePaiR-Gen (Ranjit et al. [59]) adopts retrieval-augmented generation (RAG) frameworks with pretrained ALBEF models. LLaVa-Med (Li et al. [96]) applies the LLaVa multimodal foundation model to biomedicine by employing curriculum learning on PMC-15 datasets and multiround question answering to demonstrate promising results on the VQA-RAD, SLAKE, and PathVQA datasets, showing the capacity of large pretrained multimodal models to pathology and radiology.

Even though these methods are optimized to work with macroscopic radiological scans, RAG and hallucination-reduction methods observed in X-REM and CXR RePaiR-Gen fill a significant gap in pathology text generation. These methods may achieve substantial elimination of diagnostic hallucinations by the grounding generated text using retrieved high-similarity historical WSI patches, which are pertinent on very complex and ambiguous histopathological patterns in diagnosis.

#### Specialized VLMs in pathology image analysis

Pathology presents unique challenges due to gigapixel WSIs, diverse tissue structures, and complex diagnostic criteria. In response to these issues, domain-specific VLMs have thus been developed. The visual encoder uses a pathology language image pretraining (PLIP) model trained on a large curated human pathology image text dataset, PathologyVLM, introduced by Dai et al. [97]. The method consists of 2-stage training, on domain alignment that aligns various visual domains and VQA fine-tuning on the data at WSI resolution with a scale-invariant connector that keeps resolution intact. VLM significantly outperforms both zero-shot and supervised general domain and other medical VLMs specifically designed for VQA on PathVQA and PMC-VQA datasets.

Ahmed et al. [51] developed PathAlign, based on the BLIP-2 framework, trained on over 350,000 WSI–text pairs across multiple tissue types and diagnostic categories. Image-to-text retrieval, generative report, with frozen LLMs integration, and 78% accuracy on pathologist rating without significant clinical errors are supported by the model. Additionally, this large-scale dataset and model show the feasibility of language-aligned WSI embeddings for histopathological workflows. In Huang et al. [98], hospital pathology images and accompanying text posted on medical Twitter were drawn from public sharing and synthesized into OpenPath, a dataset of over 200,000 image text pairs. Authors show that their PLIP model achieves state-of-the-art performance for zero-shot classification with a variety of external pathology datasets and largely preserves the ability to search pathology images with multimodal text queries. Authors argue that these pathology-focused models require high-quality domain-specific datasets and preserve image resolution at model levels, along with end-to-end multimodal training for clinical utility.

#### Contrasting radiology and pathology reporting paradigms

While earlier sections highlighted how radiology-based models serve as architectural blueprints for multimodal AI, it is critical to explicitly contrast the reporting paradigms of these 2 fields. Radiology reporting tends to be usually macroscopic and observational, which usually ends up in a descriptive narrative that needs to be correlated with clinical methods. In contrast, histopathology is usually the ultimate diagnostic ground truth. As a result, a direct translation of radiology-focused NLG into pathology presents a high risk of clinical risks in case pathology-specific peculiarities are overlooked.

#### Pathology-specific nuances and synoptic alignment

Pathology reporting entails unique challenges that current foundational VLMs must address. First, models have to deal with high diagnostic uncertainty and within-observer error, especially in subjective tasks such as tumor grading and definition of delicate morphological thresholds. Second, in contrast to the descriptive stories, prevalent in the radiology department, contemporary oncology is based on well-organized information. The critical prognostic modifiers, including the precise tumor margins, mitotic rates, LVI, and the outcome of additional immunohistochemical (IHC) tests, should be reflected correctly in the AI-generated pathology report [99]. Consequently, a key emerging trend in agentic AI is the transition from unstructured, descriptive text generation to the automated completion of standardized, structured reporting templates. Generative pathology models in the future should clearly be aimed at achieving the level of strict adherence to synoptic reporting, including the site entries of the College of American Pathologists (CAP) or the International Collaboration on Cancer Reporting (ICCR) [100]. The ability of VLMs to extract these particular synoptic data points of gigapixel WSIs in an automatic and faithful fashion is a basic requirement of their clinical implementation.

Synthesizing the advancements in VLMs and LLMs, a recurring architectural pattern is the necessary transition from generic biomedical text adaptation to pathology-native multimodal alignment. Initial generative models would simply bail out WSI patches into the regular radiological vision encoders, leading to a disastrous loss of cellular resolution. Reacting to this, higher-end systems have found the specialized architectural extensions now universally used, like scale-invariant connectors (as in PathologyVLM [97]) or multiscale feature alignment (as in PathAlign [51]) to preserve gigapixel context. Nonetheless, one common unreliable bottleneck of most of the reviewed systems, despite the structural advantages, is that they are dependent on training on unfiltered, image–text pairings that are noisy and uncurated based on their status on public boards or unstructured historical records. Until these models are trained on structured and synoptic level data, they will have limited ability to produce consistently clinically safe and standardized pathology reports.

### MAS and integrated frameworks in medical imaging

Recent advances in medical imaging AI have increasingly emphasized the integration of MAS and multimodal frameworks to more closely emulate the collaborative, iterative nature of clinical decision-making [101]. While single-task DL models perform the entire complex diagnostic workflow in silico, multiagent frameworks distribute the workflow across specialized, interacting AI agents for richer and interpretable medical image analysis [102]. This section covers some of the key developments in multiagent and integrated AI systems, discusses their design strategies, illustrates the benefits, and describes their clinical impact. For instance, PathFinder was designed to mimic expert pathologists’ diagnostic workflow for histopathology WSIs using a multiagent multimodal system. To address the massive size and complexity of WSIs, PathFinder relies on 4 separate agents (Triage Agent, Navigation Agent, Description Agent, and Diagnosis Agent) working in sequence to triage WSIs, navigate diagnostically relevant areas, create natural language annotations from the areas, and synthesize a final diagnosis [55]. This design shows the iterative nature of pathologists examining slides, where they look at salient patches of the image, write notes, and synthesize the information toward a cohesive clinical interpretation. PathFinder performed better than state-of-the-art models in melanoma classification by improving the final average error of pathologists by 9% and produced inherently explainable predictions represented by the natural language descriptions of the Description Agent that are qualitatively better than GPT-4o. These findings showcase the major role MAS architectures play in ensuring that the accuracy and interpretability is enhanced, which are required to be adopted by clinicians themselves [55]. Information that a given specific model is somehow qualitatively superior to GPT-4o needs to be put into context via the evaluation rubric. In such cases, the superiority was determined in terms of an LLM-as-a-judge framework that was designed with a severe 5-point Likert scale that specifically penalized models that exhibited diagnostic hallucinations and rewarded them based on clinical safety and compatibility with pathology reporting reports.

The PathGen-1.6M project also provides another example of multiagent teamwork as it is a 1.6M pathology image–text pairs creation with the help of the ensemble of specialized AI agents that create the representative patches of WSI, generate, and refine the captions that are used in the process [44]. This large-scale multiagent pipeline, as a type of big data curation in pathology AI, aims to address one of the most notable bottlenecks: the lack of well-annotated multimodal datasets, as well as the lack of African American specialists to annotate them, which is a major limitation when training powerful VLMs. The paper demonstrated how the pairs generated may be added to existing datasets to train a pathology-specific CLIP model (PathGen-CLIP) that can achieve higher accuracy on 9 zero-shot image classification tasks and on a number of tasks related to whole slide image analysis. Moreover, the authors tuned PathGen-CLIP with Vicuna LLM to produce a strong multimodal model that is optimized on pathology as a platform to next-generation hepatic diagnostic systems. This method proves the strength of multiagent models to produce and learn, at a scale and data quality which was not accessible previously. Scalable data generation and improved learning efficiency indicates that it can address a critical bottleneck in medical AI systems. 

Multiagent and integrated frameworks are becoming popular in radiology for automating report generation and diagnostic assistance. RaDialog (Pellegrini et al. [103]) includes a large vision language model that achieves clinically accurate generation of interactive dialog chest x-ray reports; the model leverages image features, structured pathology findings, and LLM capabilities. RaDialog was trained on a semiautomatically labeled instruct dataset constructed from the MIMIC-CXR database and outperformed existing models on clinical correctness while showing state-of-the-art abilities in identifying report correction opportunities and answering clinician queries. The model also significantly decreased a mean of 33% errors in comparison to prior reports and interactive models, for example, XRayGPT [103]. The dialog-based interactive nature of RaDialog reflects well in transferring to digital pathology workflow. A similar MAS in histopathology would enable automated output and would enable pathologists to pose interactive queries to a specialized visual agent regarding particular cells or regions of the cellular environment (e.g., “count the lymphocytes in this tumor microenvironment”) to establish a collaborative human–AI diagnostic cycle.

Complementing these, Biomed-DPT by Peng et al. [104] illustrates an innovative knowledge-enhanced dual modality prompt tuning approach that incorporates both text and visual prompts in biomedical VLMs. Domain-adapted text prompts and vision soft prompts are used in this method, which uses LLM-driven prompts to focus (or ignore) the model’s attention on (or areas outside) diagnostically critical regions, improving interpretability and classification accuracy. Biomed-DPT is evaluated on 11 biomedical image datasets in 3 different modalities and across 5 organs and outperforms existing prompt optimization methods by 6% to 8% in classification accuracy, providing a promising example for the applicability of combined multiagent-inspired techniques in using domain knowledge to steer AI focus and improve diagnostic accuracy [104]. The dual text-and-vision prompt tuning method of Biomed-DPT is a strategic avenue that can be used to address the huge quantity of pathology data. Devoting the output of visual agents specifically to diagnostically important tissue microenvironment (like selective playing around with isolating epithelial layers and unstated benign stroma) could be promoted by domain-specific text prompts to map this methodology to WSIs, in effect shrinking to a pivotal computational load the megapixels of image analysis.

Another LLM-based integration is presented by LEAVS, an LLM-based labeler for complex abdominal CT supervision (Lanfredi et al. [47]). LEAVS extracts structured labels of abnormalities and urgency levels for multiple organs from radiology reports using a specialized chain of thought prompting mechanism together with tree-based decision systems. LEAVS achieves an impressive average F1-score of 0.89, outperforming existing labeling tools and even human annotators on some tasks, with the capability of enriching training of vision models for the detection of abnormality across abdominal organs. When noting that systems such as LEAVS “outperformed human annotators”, it is critical to specify the study design: this claim is based on a strict quantitative comparison (F1-score) against 2 board-certified pathologists using a predefined, structured multiorgan abnormality labeling rubric, rather than an open-ended diagnostic task. This framework demonstrates how to combine an LLM-based label extraction with downstream vision models to supervise complex, multiorgan radiological data through the strength of combining text understanding and image analysis agents all in one system [47]. Although LEAVS was designed to supervise multiorgan CT, the chain of thought prompting decision tree logic is very relevant to the multistain of pathology. A text-parsing agent would be able to interact with complex biopsy texts to produce structured, weakly supervised labels that allow the training of downstream WSI vision with minimal or negligible supervision that requires pathologists to make pixel-level annotations.

Innovations in report generation via cross-modal representation learning are exemplified by the pathological clue-driven representation learning (PCRL) model of Zheng et al. [105], which addresses 2 major challenges in brain CT report generation: redundant and shifted visual representations. PCRL builds enriched cross-modal features that align better with clinical report semantics by constructing pathological clues from segmented regions as well as pathological entities. This unified framework provides for a smooth transfer, from feature representation to report generation, via fine-tuning of LLMs within task-specific instruction. Experimental results show PCRL’s state-of-the-art performance and prove to be a meaningful advancement to MAS that fuse radiological clinical knowledge tightly with visual data representation for radiological reporting [105]. The PCRL of building pathological clues readily induced by the segmented radiological sites could be readily transposed into frameworks of proclaiming histopathology. One way a pathology MAS would achieve this would be to run a tissue-segmentation agent (e.g., to extract glomeruli in kidney biopsies) before running the text-generation agent to assure that its text reports are strictly limited to localized cellular deviations.

Subsequently, the PathologyVLM by Dai et al. [97] leverages a 2-stage training method, consisting of domain alignment from pathology image text datasets and then end-to-end fine turn-on VQA tasks. PathologyVLM achieves strong cross-pathology performance on both supervised and zero-shot VQA tasks by employing a PLIP model as the visual encoder and a scale-invariant connector to avoid information loss due to image resizing. The core of this success is the scientific significance of domain-specific multiagent inspired components of computational pathology; the domain-specific design enables one to keep more of the details of the pathological images intact in a better way [97]. ChatEXAONEPath (Kim et al. [106]) can also be trained on expert-level multimodal LLMs anywhere on top of WSIs and similar histopathology reports of the TCGA based on a retrieval-based data generation pipeline and AI-based evaluation protocols. Combining multiagent strategies in multimodal data, such as ChatEXAONEPath to assist clinicians with complicated cancer diagnosis, has an acceptance rate of 62.9 on pan-cancer WSIs diagnosis [106].

Taken together, these multiagent and integrated systems constitute the medical AI paradigm shift to no longer isolated task-specific frameworks, to collaboratively context-aware models that are more appropriate to clinical processes. These systems enhance accuracy, efficiency, and understandability by coordinating specialized agents. They are also able to generate and annotate data in a scalable way, which is a major issue to deal with in medical AI studies. The future will be an area of further clinical integration, agent communication, and wide-range multimodal fusion—eventually aiming at AI systems that can improve clinical decision-making via transparency and adaptability as well as reliability.

#### Detailed multiagent pipelines: PathFinder and SlideSeek

The recent developments have shifted the MAS into a theoretical framework to practical, pathology-specific pipelines with the capability of performing autonomous diagnostic argument. The most notable instances of such development are the PathFinder and SlideSeek frameworks that use different agent designs to replicate the process of a human pathologist.

##### Agent roles

PathFinder is a program based on a dedicated 4-agent architecture that can process WSIs in order to extract features and processes information to calculate outputs in sequential order in real time [55]. The process starts with a Triage Agent that first undergoes a low-magnification examination to categorize the WSI as the benign or risky. In case it is considered risky, the Navigation Agent could simulate the behavior of a panning and zooming gesture of a pathologist to locate ROIs and create an importance map. This guides the Description Agent that employs a VLM to write natural language descriptions of the localized cellular structures. Lastly, a Diagnosis Agent combines these mass descriptions to come up with a concrete diagnostic description.

In contrast, SlideSeek utilizes a hierarchical, reasoning-enabled structure built upon the PathChat+ foundation model [56]. It is based on a Supervisor Agent that serves as an overall coordinator. First hypotheses in high-level WSI overviews are formulated by the supervisor who then distributes single exploration tasks to a number of Explorer Agents (or pathologist agents). These explorers will explore the assigned ROI coordinates at the same time, simultaneously extract morphological features, and project them back to the supervisor. After the supervisor concludes that diagnostic evidence has been sufficiently collected, a different Reporting Agent puts the data together as interpretable and visually based diagnostic summary.

##### Communication protocols

The effectiveness of these systems hinges on their underlying communication protocols. PathFinder is based on a protocol that is sequential, state-passing with the output of one agent triggering and constraining the action of the subsequent agent (e.g., the visual importance map of the Navigation Agent being the direct determinant of the set of text that the Description Agent can generate) [55].

SlideSeek, on the other hand, employs a hierarchical “hub-and-spoke” protocol. Explorer Agents are not able to communicate with each other but rather run parallel and are able to communicate only with the central Supervisor Agent. The Explorer Agents perform the work in parallel scanning the slide at the given level of magnification and recording the morphology of the tissue in their respective reports to the supervisor with the assistance of PathChat+. The managerial aspect of this centralized state means that the supervisor can make a series of adjustments of the overarching diagnostic plan by referencing incoming parallel streams of data until an evidence level is achieved [56].

##### Failure modes

Although medical MAS architectures are highly designed, they are all prone to special collaborative failure modes. The recent study of auditing medical multiagent collaboration reveals the following vulnerabilities that are critical:

• Echo-chamber amplification (flawed consensus): In the event that an upstream agent (such as the Description Agent in PathFinder) had hallucinated a morphological feature or incorrectly interpreted visual evidence based on constraints of the base model, downstream agents can be confident and hasty about their error without checking it. This causes a bad judgment in that the eventual diagnosis is sure to be incorrect.
• State synchronization failures: In hierarchical systems such as SlideSeek, failure of the central supervisor to correctly update the state of his/her hypothesis, in response to the explorer, can cause stale state propagation or state update conflicts, which can propagate duplicate work or give conflicting diagnostic information.
• Infinite loops (circular task delegation): It is possible to endlessly get stuck in an unrestricted back-and-forth loop of agents without strict termination conditions. As an example, a supervisor agent could constantly demand finer-grained analysis of ROI of the Explorer Agents but will, in no manner, arrive to the diagnostic confidence level necessary to cause the reporting agent to activate.

### Comparative analysis of state-of-the-art multimodal, agentic, and benchmarking frameworks

Recent advancements in the rapid development of computational pathology have brought the new wave of multimodal-based foundation models, autonomous multiagent pipelines, and strong clinical metrics. Table 1 is a synthesis of these state-of-the-art systems in tabular form, to indicate the architectural design of the systems and their main performance results in order to show us how fast the field is transforming.

**Table 1.** Comparison of the recent state-of-the-art multimodal and multiagent pathology frameworks

Ref.	System	System type	Dataset scale	Supervision paradigm	Clinical reader validation	Evaluation metrics	Key features	Key results
[56]	PathChat+ and SlideSeek	MLLM and multiagent	1.13M instruction samples, 5.49M QA turns	Instruction-tuned	Yes	VQA accuracy, DDxBench accuracy	Instruction-tuned pathology MLLM. Hierarchical agent framework (supervisor/explorer) for ROI navigation.	Superior diagnostic reasoning on gigapixel WSIs. Highly interpretable, autonomous evidence gathering.
[46]	TITAN	Foundation model	335,645 WSIs, 423k synthetic captions, 183k reports	Visual SSL	Yes	Zero-shot/Few-shot Classification, Cross-modal retrieval	Pretrained on more than 335,000 WSI–report pairs. Utilizes visual self-supervised learning and vision-language alignment.	Exceptional zero-shot and few-shot classification. High accuracy in cross-modal retrieval without fine-tuning.
[124]	SlideChat	Vision-language assistant	4.2k WSI–caption pairs, 176k VQA pairs	Visual instruction learning	Yes	BLEU-1, GPT score, VQA accuracy	Processes entire gigapixel WSIs simultaneously. Employs a sparse-attention slide-level encoder to retain global context.	Outperforms standard patch-based models on WSI-level VQA.
[52]	PMPRG	Report generation	7,422 WSIs (multiorgan dataset)	Contrastive SSL (MR-ViT) + language generation loss	Yes	METEOR, BLEU, ROUGE, clinical efficacy (CE)	Generates structured, patient-level, multiorgan reports. Guided by predefined clinical tags and multiscale visual features.	Significant improvements in CE metrics and diagnostic tag classification accuracy.
[51]	PathAlign	Report generation	>350,000 WSI and diagnostic text pairs	Vision-language alignment (BLIP-2)	Yes	Pathologist acceptance rate (78%), classification accuracy	Aligns multiscale WSI features with clinical text. Evaluated using rigorous pathologist grading schemes.	High pathologist acceptance rates. Demonstrates a low rate of clinically significant errors or omissions.
[55]	PathFinder	Multiagent pipeline	M-Path skin biopsy dataset (238 cases)	Binary cross-entropy + text-conditioned visual navigation	Yes	Diagnostic accuracy (74%), F1-score	4-agent sequential workflow (triage, navigation, description, diagnosis). Mimics human pathologist panning/zooming behavior.	Outperformed SOTA models in melanoma classification by 8%. Beat human pathologist average accuracy by 9%.
[125]	PathMMU	Benchmark	33,428 QA pairs, 24,067 images	N/A (evaluation benchmark)	Yes	Zero-shot accuracy, human–expert comparison	Largest expert-validated multimodal benchmark in pathology. Contains > 33,000 QA pairs testing visual grounding and reasoning.	Revealed a significant performance gap: current top LLMs still lag considerably behind expert pathologists.
[108]	PathBench	Benchmark	15,888 WSIs from 8,549 patients	N/A (evaluation benchmark)	None	Bootstrapped 95% CIs, Wilcoxon signed-rank test, ranking score	Comprehensive, multicenter, multitask evaluation framework. Implements strict data leakage prevention.	Standardized model evaluation, highlighting current generalization gaps across diverse clinical environments.

MLLM, multimodal large language model; QA, question answer; VQA, visual question answering; ROI, region of interest; WSI, whole-slide imaging; SSL, self-supervised learning ; BLEU-1, bilingual evaluation understudy ; GPT, generative pre-trained transformer ; PMPRG, patient-level multiorgan pathology report generation; BLIP-2, bootstrapping language-image pre-training ; SOTA, state of the art ; LLM, large language model; CI, confidence interval

## Discussion

### Overview of results

In this section, a synthesis of the findings of the most relevant studies reviewed in this SLR is provided on the topics of DL methods, VLMs, and LLMs, as well as on integrated multimodal systems and MAS being used in the analysis of pathology images and in the generation of medical reports. These studies represent various innovative approaches, including CNNs and transformer models used in the image segmentation and classification task, as well as advanced multimodal approaches, which integrate visual and textual data to provide increased support in diagnosing and to generate reports automatically.

The findings demonstrate high progress across various domains, including the increased segmentation accuracy on pathology images, strong zero-shot classification on VLMs, and improved diagnostic decision-making with multiagent collaboration. Nevertheless, there are issues of data generalizability and incorporation of domain knowledge as well as explainability. Taking into account these summarized results allows one to reach certain conclusions about this modern situation, where an AI-based pathology analysis can thrive , and provide some suggestions about the direction that the future research and clinical implementation should take. Tables 1, 2, and 3 summarize the approach and the conclusions of notable representative studies in these 3 fields.

**Table 2.** Deep learning techniques used in pathology

Ref.	Year	Methodology	Results
[41]	2016	Used CNNs (Caffe framework) for nuclei, epithelium, tubule segmentation, mitosis detection	Achieved F-scores between 0.53 and 0.90 on various segmentation/detection tasks
[42]	2023	Developed HipoMap to convert WSIs into structured maps + CNNs for lung cancer classification	AUC 0.96 for lung cancer classification; improved survival analysis with TCGA data
[62]	2024	Integrated U-Net and EfficientNetV2 with novel heat map generation algorithm	Precision 92.86%, recall 86.67%, processing time 8.26s per image; high interpretability
[64]	2023	CNN-based risk stratification model for oral leukoplakia cancer progression	High-risk group 4× more likely to develop cancer; strong prognostic value
[63]	2023	Cycle-consistent GAN for synthetic data generation; modified U-Net for nuclei segmentation	DSC improved from 0.805 to 0.984 with synthetic images; accuracy 0.97
[66]	2023	Diversity-aware data acquisition function and model simplification for tumor-infiltrating lymphocytes classification	Improved model performance and 4.3× faster active learning execution
[67]	2021	Attention-based weakly supervised learning (CLAM) on WSIs with slide-level labels	Superior data efficiency and adaptability; interpretable subregion identification
[43]	2025	EAGLE framework with tile selection (CHIEF) and feature extraction (Virchow2)	Outperformed SOTA models by up to 23%; 99% faster processing (2.27 s/slide)

CNN, convolutional neural network; WSI, whole-slide imaging; AUC, area under the curve; TCGA, The Cancer Genome Atlas; GAN, generative adversarial network ; DSC, Dice similarity coefficient; CLAM, clustering-constrained attention multiple-instance learning ; EAGLE, Efficient Approach for Guided Local Examination ; CHIEF, clinical histopathology imaging evaluation foundation ; SOTA, state of the art

**Table 3.** Vision-language models and large language models in medical imaging

Ref.	Domain	Year	Methodology	Results
[103]	Radiology and mixed medical modalities	2023	Vision-language integration with LLMs, fine-tuned on chest x-ray reports (MIMIC-CXR)	33% improvement in report correction; superior clinical correctness and interactive abilities
[44]	Pathology modalities (whole-slide imaging)	2024	Multiagent collaboration to generate pathology image-caption pairs; train pathology-specific CLIP	Significant improvement across 9 zero-shot tasks and WSI analyses
[97]	Pathology modalities (whole-slide imaging)	2024	Two-stage learning: domain alignment + VQA fine-tuning; pathology-specific visual encoder	Outperformed general domain and medical VLMs on VQA tasks; better image feature preservation
[96]	Radiology and mixed medical modalities	2024	Fine-tuned LLaMA model with medical multimodal instruction-following datasets	Demonstrated strong VQA capabilities on multiple biomedical datasets
[98]	Pathology modalities (whole-slide imaging)	2023	Trained PLIP multimodal model on OpenPath dataset (208k pathology images with captions)	Achieved F1-scores 0.565–0.832 on zero-shot classification; improved knowledge retrieval
[106]	Pathology modalities (whole-slide imaging)	2024	Retrieval-based pipeline using WSIs and TCGA reports; evaluation on pan-cancer diagnosis	62.9% acceptance rate in clinical diagnosis; capable of pan-cancer WSI understanding

LLM, large language model; MIMIC-CXR, Medical Information Mart for Intensive Care-Chest X-ray ; CLIP, contrastive language image pre-training ; WSI, whole-slide imaging; VQA, visual question answering; VLM, vision-language model; LLaMA, large language model Meta AI ; PLIP, pathology language image pretraining; TCGA, The Cancer Genome Atlas

### Critical appraisal and evidence gaps

Although the literature has shown high architectural developments in multimodal and agentic system of pathology, a critical criticism on the evidence underpinnings has established a high level of gaps in methodological development as well as validation. Most of the researches nowadays state excessively positive outcomes by confusing statistical significance with clinical significance, and hence, more strict examination of their real deployability is required.

#### Dataset leakage and evaluation protocols

A primary evidence gap lies in the structural evaluation protocols of massive VLMs and LLMs. A number of studies state extraordinary zero-shot capabilities, although such conclusions are often undermined by silent leakages of datasets [43,46]. Since foundation models are being trained on large, usually low-quality documented corpora on the internet, there is a possibility that public benchmark test sets (like those derived in TCGA) have been accidentally represented in the training AI pitfalls data. In the absence of serious, verifiable isolation of multicenter, out-of-distribution test sets, the values of AUC and accuracy values presented in most current papers are likely inaccurate reflections of the generalization properties of the models being represented.

#### Quality of clinical validation

Furthermore, the quality of clinical validation across the reviewed literature is highly variable. While evaluating generative outputs requires “human-in-the-loop” reader studies, many frameworks rely on critically underpowered validation setups, often utilizing only 1 or 2 pathologists to grade the AI’s output [47,51]. In order to develop a true clinical trust, the next research should provide metrics of formal interobserver agreement (e.g., Cohen and Fleiss Kappa) with a larger group of specialists certified by the board. There is further a preponderance of literature to represent statistical significance as clinical significance and vice versa. The average increase in a classic NLG score such as METEOR of 2% could be statistically significant but clinically insignificant once a model continues to make sporadic hallucinations that a patient has a malignancy.

#### Clinical maturity and technology readiness

Finally, it is necessary to contextualize the maturity of these systems using a technology readiness level (TRL) framework. The large proportion of the multiagent and multimodal system reviewed in this paper have TRL 3 (experimental proof of concept) to TRL 5 (in a simulated or relevant laboratory context) performance. They are not implementable clinical tools. The inability to expressly distinguish between a well-controlled proof of concept and an adequately sound regulatory-tarted system (TRL 8 to 9) generates an illusion of clinical preparedness, in the sphere. To cover this gap, the change in the previous testing of retrospective, well-curated datasets would have to change to a prospective, real-world clinical trial [107].

### Addressing the research questions

CNNs in addition to transformer-based architecture have shown major improvements in the analysis of pathology images, with the advancement of the DL models. These models are effective in addressing the high dimensionality and the heterogeneity of WSI, which exploits the hierarchical feature extraction and attention scheme of these studies [41,42]. The U-Net-based EfficientNetV2 hybrid models further boost the segmentation accuracy at decreased computational costs [62]. Yet, variability in staining procedures, imaging protocols, and data from many clinical settings remain barriers to model generalizability and robustness [90]. To lessen annotation burden and increase model adaptability, strategies such as AL and weakly supervised approaches are introduced [66]. Table 2 provides a summary of DL techniques used in pathology.

#### How have VLMs and LLMs been employed to generate clinically relevant text from medical images, particularly in pathology?

An overview of VLMs and LLMs used for pathological image analysis is given in Table 3. VLMs and LLMs have shown promising capabilities in bridging the gap between image analysis and automated report generation. PathGen-1.6M and RaDialog serve as examples of exploiting visual data in conjunction with textual clinical knowledge to generate coherent and clinically relevant reports from pathology images and radiological scans [44,103]. Fine-tuning vision encoder and language models with large image datasets of caption pairs for domain-specific tasks improves diagnostic accuracy and reduces the time for report generation [97]. These advances notwithstanding LLMs remain limited in terms of domain specificity to medical knowledge, context understanding, and potential for complex/rare cases, thus requiring future work with clinical ontologies and real-world data [94].

#### What roles do MAS play in integrating image analysis with text generation in medical diagnostics, and what are the architectures and strategies that facilitate effective interagent collaboration?

Multimodal systems and MAS in medical imaging are described in Table 4 along with the adopted methodology and their associated results. MAS provide a promising paradigm for collaborative AI in pathology by orchestrating specialized agents to perform distinct tasks such as image triage, feature extraction, report drafting, and validation [47]. Collaborative AI in pathology shows promise when implemented as a MAS that coordinates agents specializing in different tasks (image triage, feature extraction, report drafting, and validation) as in PathFinder. For example, PathFinder provides a demonstration of how agents can iteratively traverse WSIs and produce interpretable diagnostic explanations that outperform pathologist performance in melanoma classification [55]. Unlike monolithic models, MAS architectures are more scalable, interpretable, and modular to equip more flexible, more clinically oriented diagnostic workflows [92]. Yet, there are existing challenges in unifying agent-to-agent communication, guaranteeing agent output consistency, and combining agent outputs with current clinical decision-making processes [105].

**Table 4.** Multimodal and multiagent systems in medical imaging

Ref.	Domain	Year	Methodology	Results
[55]	Pathology modalities	2025	Multiagent system with triage, navigation, description, and diagnosis agents	Outperformed SOTA in melanoma diagnosis by 8%; surpassed pathologists’ average by 9%
[44]	Pathology modalities	2024	Multiagent collaboration for scalable pathology image–text pair generation	Enabled training of powerful pathology VLMs with improved zero-shot and WSI task performance
[103]	Radiology and cross-domain modalities	2023	Vision-language integration + interactive dialog system with specialized instruction dataset	33% improvement in report correction; strong clinical report generation performance
[104]	Radiology and cross-domain modalities	2025	Knowledge-enhanced dual prompt tuning using LLM-driven domain prompts and vision soft prompts	Classification accuracy improved by 6%–8% over prior methods on 11 biomedical datasets
[47]	Radiology and cross-domain modalities	2025	Chain-of-thought prompting with decision-tree system for extracting structured labels from reports	Achieved F1-score 0.89; outperformed human annotators in multiorgan abnormality labeling
[105]	Radiology and cross-domain modalities	2024	Pathological clue-driven cross-modal representation learning with LLM fine-tuning	SOTA performance in brain CT report generation; improved cross-modal consistency
[106]	Pathology modalities	2024	Multimodal LLM integrating WSIs and histopathology reports with retrieval-based data generation	Effective pan-cancer diagnostic understanding; clinical evaluation acceptance 62.9%

SOTA, state of the art ; VLM, vision-language model; WSI, whole-slide imaging; LLM, large language model; CT, computed tomography

### Clinical applicability and practical considerations

While many DL and multimodal models have demonstrated promising results in controlled research settings, few have reached widespread clinical deployment. However, these fast and accurate models, including EAGLE and RaDialog, are still moving toward real-time clinical application, with remaining integration barriers, for example, the use of appropriate computational infrastructures and validation in alignment with diverse patient populations [43,103]. Furthermore, the datasets in training often lack representation of all the demographic groups, which can influence clinical generalizability [44]. This remains a key requirement for AI adoption and interoperability with hospital information systems, EHRs, and the laboratory workflow still necessary. LEAVS and PathAlign focus on structured label extraction and reporting to be aligned with clinical terminology and therefore easily connected to medical experts [47,51]. Pathology workflows are complex and variable across institutions; therefore, they require adaptable AI frameworks that can be configured to local standards [98].

The challenges related to privacy and the compliance with the regulations are critical issues when it comes to the implementation of the AI systems that utilize patient data. Data anonymization and federated learning solutions develop as potential solutions to overcome privacy concerns without endangering model performance [46]. Secondly, the elements of ethics, such as bias alleviation, transparency, and accountability, become necessary when the AI-assisted decisions may be accompanied by possible clinical effects [51]. Multiagent frameworks like PathFinder and LEAVS offer explainable reasoning and confidence estimate, in which clinicians can learn to trust and cooperate with the help of human agents in the loop [47,55]. To ensure that AI results match clinic expectations and workflows, training and engagement of pathologists in the system development is required [44].

#### Defining clinical deployment readiness

Although a good number of the studies reviewed show outstanding retrospective performance, the transition between a controlled laboratory environment and the clinical real-life environment needs rigorous, verifiable evidence. In support of the fusion of theory with practice, we suggest a standardized “Clinical Deployment Readiness Checklist”. This checklist is a synthesis of the required operational specifics, minimum deployment evidence, and integration specifications that the next generation of computational pathology frameworks that need to be deployment-ready must report. Table 5 provides a deployment readiness checklist for pathology AI in the context of clinical applications.

**Table 5.** Clinical deployment readiness checklist for pathology AI

Readiness criteria	Verifiable item/Minimum evidence required	Rationale for pathology
External validation	Testing on independent, multicenter cohorts completely unseen during training.	Ensures the model generalizes across different hospital populations and laboratory protocols.
Subgroup and stress analysis	Performance stratified by WSI scanner type, magnification levels, and staining variations (e.g., H&E vs. IHC).	Pathology images are highly susceptible to color and hardware domain shifts; models must prove robustness to these artifacts.
Calibration metrics	Reporting expected calibration error (ECE) or Brier scores alongside accuracy.	Clinicians must know if the model’s confidence scores accurately reflect the true likelihood of a diagnosis.
Compute and latency	Explicitly stating VRAM requirements, inference time per gigapixel WSI, and hardware specifications.	Many hospital IT infrastructures cannot support massive GPU clusters; operational latency must fit standard diagnostic workflows.
Integration touchpoints	Demonstrated compatibility with standard Laboratory Information Systems (LIS) or WSI viewing software (e.g., DICOM-WSI standards).	Standalone, fragmented AI software disrupts pathologist workflows; seamless LIS integration is mandatory for adoption.
Audit logging and traceability	Mechanisms to log the specific WSI patch or text prompt that triggered a specific diagnostic output.	Essential for legal compliance, regulatory audits, and retrospective failure analysis if the AI makes an error.

AI, artificial intelligence; WSI, whole-slide imaging; H&E, hematoxylin and eosin; IHC, immunohistochemical; VRAM, video random access memory ; IT, information technology; GPU, graphics processing unit

When applying this deployment readiness checklist to the state-of-the-art frameworks reviewed in this study, a clear gap emerges between experimental success and true clinical deployability. Although there is no single system that meets all the requirements, some of the pioneering frameworks show high compliance with certain areas. As an example, in compute and latency, since the EAGLE framework [43] deals with hardware constraints, processing time is minimized to about 2.27 s per gigapixel WSI. Regarding external validation and cross-center testing, traditional foundational models such as TITAN [46] or models pretrained on the OpenPath data [98] have been shown to have strong zero-shot generalization through the dissimilar and hidden cohorts. In the case of audit logging and traceability, an audit trail is a natural by-product of multiagent pipelines like PathFinder [55] and SlideSeek [56], where the sequential spatial coordinates and texts at a local position are recorded by the navigation agents. Furthermore, frameworks such as PathAlign, integrate rigorous pathologist-led studies to ensure that the outputs are clinically meaningful and well aligned with real-world diagnostic standards [51]. There are, however, critical voids that exist in the literature in general, especially in the areas of calibration metrics and organized integration touchpoints with Laboratory Information Systems (LIS). Meanwhile, benchmarking systems such as PathBench [108] will be necessary in implementing these multicenter, deployment-centered appraisals.

### Challenges

One of the most prominent challenges in the application of AI technologies in medical imaging is the generalizability of models across different clinical environments. CNNs and VLMs, in many cases, are trained on specific datasets that come from some set of particular institutions or imaging protocols. Therefore, these models can work well if used on their training data but have difficulty when used on other institutions’ data that come from different equipment, imaging approaches, or patient populations [109]. There is a lack of generalization because of variability in the type of scanners used, in the staining procedures for histopathology, and in the acquisition protocols. For example, a slide scanner at one hospital might be a different model than a slide scanner at another hospital, or one hospital might prepare tissues using a different method than another hospital, and this can result in variations in the quality of the images received [45]. Due to a lack of consistency in data across clinical settings, the performance of the AI models degrades, and therefore, the AI models become unreliable for deployment to real-world settings where the data are more diverse and unpredictable. To address this challenge, we need domain adaptation strategies to train AI models to adapt to the inherent variability of clinical datasets and generalize to different clinical settings such that they are able to provide accurate results across different clinical settings [110].

The interpretability and explainability of AI models are another significant challenge that hampers their acceptance and adoption in clinical practice. CNNs and VLMs have demonstrated impressive performance, and many clinicians regard these as black boxes. Although the initial methods of XAI are based on the traditional saliency maps and generic mechanisms of attention, recent studies prove to be untrustworthy in sophisticated medical scenarios. Indicatively, research comparing saliency map techniques such as Grad-ECLIP has shown that conventional saliency maps do not deliver clinically meaningful localization, and more often tend to identify background pixels that are not spurious but actually present embedded pathologies in the image of interest instead of tracking them down and raising their salinity score [111]. The situation is even more complex with multimodal VLMs; to be able to explain anything accurately, token-to-patch grounding must be very accurate. To foster trust, XAI approaches should not be limited to high-level heatmaps whereby a particular generated clinical term (such as nuclear atypia) is anticipated to project to the right cellular structure in the gigapixel WSI in a consistent and reliable manner. Researchers are working on steps to ensure explainability by developing XAI models such as attention systems and saliency maps used to identify the feature of the image the model used to make a choice [112].

Data scarcity and annotation challenges present another major obstacle in the deployment of AI in medical imaging. WSIs as high-resolution pathology images demand extensive annotations from domain experts, e.g., pathologists, to effectively train DL models. However, the process of annotating these images is expensive and time-consuming due to the very specialized skill set required for the job and the time it takes to do it [113]. Additionally, there is a dearth of available annotated data, and it is very difficult to train strong AI models with the dataset if the disease being studied is rare or the tissue type is unusual. The annotated data are also often not representative of the full range of variability seen in real-world clinical environments, even if there exists some annotated data. For example, data used for training may derive from a limited body of patients or a single institution, and the data are not diverse enough. Furthermore, patients' population diversity, e.g., ethnic minorities, ) is poor, which further compounds this issue since AI models trained only on a certain demographic may appear biased toward that demographic [114]. An absence of diverse datasets can create poor-performing AI systems on populations that are underrepresented and subsequently biased diagnostic outcomes. To make progress on this challenge, larger and more diverse datasets must be created to train simpler models or learn from smaller datasets or unannotated datasets (synthetic data generation and few-shot learning) [115].

In addition to generalizability and data scarcity, the predisposition of VLMs and LLMs to hallucinate remains one of the greatest clinical safety obstacles. Whereas general medical AI hallucinations tend to have anatomical findings of fabricated claims, pathology poses a special and highly organized system of hallucination threat. These pathology-related hallucinations could be generally grouped into 3 different types:

• Fabricated grading and subtyping: Generative models can end up giving a false certainty assigned to a given tumor grade (such as Nottingham grading in breast cancer) or histological subtype based on spurious statistical associations instead of actual visual evidence of mitotic counts or nuclear pleomorphism [116].
• Omission of prognostic modifiers: Models can produce syntactically competent descriptions of a tumor, and be entirely silent with regard to whether or not LVI, perineural invasion, or surgical margins are clear, which determine the kind of treatment to be given after surgery [117].
• Hallucinated ancillary testing: A hallucination of one of the most perilous forms is that a model can create its own IHC or molecular output (e.g., pronouncing a tumor as HER2 positive) by just using standard hematoxylin and eosin morphological priors, which are even unnecessary to actually carry out.

Because pathology reports serve as the definitive ground truth for oncology workflows, these hallucinations are not merely technical errors but severe patient safety threats. To reduce these risks, it is necessary to abandon unconstrained and free-text generation. Some effective strategies would be to base the generated text on visual evidence through high-fidelity MIL attention maps, to anchor outputs on tested historical patches with RAG [58,59] to force the model to produce text in standardized synoptic reporting templates (CAP or ICCR guidelines) in order to eliminate the important clinical aspects [99,100].

### Future directions

Although the general progress of AI is vast, computational pathology directions in the future need to be prioritized explicitly, taking into account the individual bottlenecks of gigapixel WSIs. The large size, context dependence, and staining variability of WSIs demand different operational habits than those of macroscopic radiology. We propose the following prioritized roadmap to translate current AI capabilities into clinical practice:

• Priority 1: High-fidelity multimodal explainability: Going beyond generic, unreliable heatmaps, the future operational models will need to be implemented with VLM-specific XAI methods (like Grad-ECLIP algorithms) to ensure spatial and semantic faithfulness. The most important requirement needed to be granted regulatory approval is to ensure the generated diagnostic text is explicitly anchored on verifiable WSI tissue morphology.
• Priority 2: WSI-optimized federated learning: Due to the scale of pathology WSIs—gigapixels—their standard federated learning protocols between small medical images (such as x-rays) cannot be computed in practice. The future architectures should be made absolutely optimized with the transfer of ultra-high-resolution data and complex domain adaptation of a variety of multicenter laboratory staining protocols.
• Priority 3: Standardizing multiagent operational protocols: To translate the MAS into clinical practice, it will be necessary to enforce standard communication and operation protocols. Subsequent studies should specify hard regulatory guardrails of autonomous multiagent diagnostic loops, the standard format in which specialized agents (e.g., Triage, Navigation, and Description Agents) transfer coordinates and hypothesis states without going into a defensive loop or hallucination echo-chambers.

Further studies will be needed to address the problem of generalizability in dissimilar datasets and clinical settings by creating stronger and more flexible AI applications. One such method is called federated learning, which allows collaborative (to be more precise, decentralized) model training on many datasets without requiring the sharing of sensitive patient data, which is a promising research that is currently underway to be implemented in research practice [118]. This achieves the exposure of models to a wide variety of data in various practice settings without loss of patient privacy. Through federated learning, models can learn on data at (different) institutions without ever having to have a patient transfer among them in the healthcare setting where data privacy is of paramount consideration. Since not every dataset involved in federated learning is generated by the same organization, federated learning may enable AI models to correct the absence of training data on the data of a single organization and achieve higher results on other datasets and platforms [119].

Moreover, data to harmony methods such as color normalization and image resampling may be utilized to reduce any variations related to the use of various imaging protocols and enable models to be trained on data of different origins to be compared and implemented in a clinically significant manner across sites. These guidelines on the scalability and resilience of AI models in medical imaging pave the way for practical applications of these models in real-world clinical settings [120].

Another key area for development is the explainability and interpretability of AI systems in medical imaging. As the adoption of AI in clinical decision-making is more completely incorporated, it is imperative that these models not only provide the correct answer, but also clarify with clear and understandable processes how and why they obtained the correct answer in the first place; it is vital that such models ought not only to provide correct answers, but also to clarify the manner in which they do so, and the reasons behind it [112]. To ensure that clinicians are comfortable enough to use the model to make their decisions in patient care, they must feel at ease with the logic used by the model in making their predictions. This desire has led to the active advancement of XAI methods. The other method is the one that applies attention-based mechanisms, which means that in making the predictions, the AI model paid attention to the parts in the image (regions of an image) that it was interested in. That is, it makes us realize why an approach of models has categorized a region of a tissue this way and why a model is focusing on the right aspects of the image. In addition, the use of XAI with clinical ontologies (i.e., structured sets of clinical terms) will improve the interpretability of the AI systems as they will be explained by an existing body of knowledge and clinical practices that are already known about medical conditions and their treatment [20]. Moreover, such systems can also be made explainable and clinically usable through the human-in-the-loop system architectures, where clinicians can communicate with the AI models to refine the diagnoses and reports, which becomes not only accurate, but also clinically actionable as well as explainable and knowledgeable by humans working with the models [121].

Finally, the issue of data scarcity and bias can be addressed through the development of large-scale, diverse, and representative datasets that include data from various demographic groups and clinical environments. The AI models that are trained today for medical imaging are frequently trained on datasets that are not sufficiently diverse, leading to biased and nongeneralizable outcomes for all patient populations [121]. Crowdsourced datasets are being used to gather large amounts of annotated medical data from a wide range of collectives, including diverse patient groups and institutions to combat this. Transfer learning and unsupervised learning techniques may also make it possible to employ similar models in new tasks with less labeled training examples. AI models can be trained to perform well across different clinical environments and patient populations by using domain adaptation techniques that help improve models’ generalizability [122]. Additionally, synthetic data generation methods could be leveraged to create artificial data that could be used in conjunction with empirical datasets to cater to the lack of large and underrepresented datasets. Training AI systems on diverse and high-quality datasets, including the appropriate input for the patient, is important to ensure they are fair, accurate, and clinically applicable so that patient outcomes are improved [123].

To overcome the safety barriers posed by hallucinations, future research must prioritize rigorous, domain-specific evaluation frameworks and measurable mitigations. NLP measures that are generalized cannot measure the clinical severity of fabricated medical findings. Rather, the profession needs to embrace the use of specialized benchmarking instruments like Med-Hallmark that present hierarchical scoring schemes (e.g., MediHall Score) as a means of establishing the precise clinical severity and underlying type of multimodal medical hallucinations. Equally, testing explicitly on multimodal reasoning in microscopy with forms of MicroVQA can give results in which researchers can systematically isolate and quantify the errors of perception as opposed to errors of reasoning-induced hallucinations. Secondly, it will be needed to develop methods that will ensure general completeness of the facts and cross-modality of structured medical reporting, including the strategies mentioned by Moll et al. By integrating rigorous safety standards and grounding mechanisms into the development life cycle, future pathology MAS can ensure that their outputs remain closely aligned with the underlying visual evidence.

#### Standardizing LLM-as-a-judge rubrics for pathology

As the evaluation of generative medical AI increasingly relies on “LLM-as-a-judge” frameworks, the lack of standardized rubrics makes cross-study comparisons highly subjective. Future studies must adopt and report a transparent, standardized scoring rubric to ensure reproducibility. We propose that future evaluations report against the following core dimensions:

1. Factual accuracy (0 to 5): Does the generated text correctly identify the primary diagnosis, histological subtype, and grade present in the ground-truth data?
2. Clinical completeness (0 to 5): Does the output include all necessary secondary prognostic markers (e.g., margins, LVI, and mitotic rate)?
3. Safety and hallucination penalty (−5 to 0): Are there any fabricated morphological findings or contradictory statements that could lead to patient harm? Severe hallucinations should result in an automatic failure for the prompt.
4. Reasoning alignment (0 to 5): Is the diagnostic conclusion logically supported by the described visual evidence, mimicking a pathologist’s step-by-step deductive process?

## Conclusion

This SLR has explored the evolving landscape of DL, VLMs, LLMs, and MAS as applied to pathology image analysis and automated report generation. To address these challenges in high-resolution whole slide images, DL approaches have shown remarkable success in performing accurate segmentation, classification, and feature extraction. At the same time, VLMs and LLMs have been integrated to interpolate between visual data and clinical text to generate clinically meaningful diagnostic reports. This integration is also further enhanced by MAS, which organize specialized AI agents to complete complex tasks together, improving diagnostic accuracy, scalability, and interpretability. Although major progress has been made, challenges remain, including model generalization, interpretability, data standardization, and ethical issues, that continue to preclude widespread clinical adoption.

Looking ahead, the future of pathology image analysis lies in developing robust, scalable, and clinically aligned hybrid frameworks that leverage the strengths of DL, VLMs, LLMs, and multiagent collaboration. To build trust and move toward integration in real-world clinical workflows, addressing research gaps that these techniques introduce through the use of larger, more diverse datasets, improved model explainability, and privacy-preserving training methods will be critical. The convergence of these leading-edge AI technologies promises to revolutionize pathology diagnostics through the automation of labor-intensive tasks, reducing human error and hopefully ultimately leading to improved patient outcomes. This review provides a foundational roadmap to guide ongoing research and development toward the realization of fully integrated, AI-driven pathology systems.
